# *scoreInvHap*: Inversion genotyping for genome-wide association studies

**DOI:** 10.1371/journal.pgen.1008203

**Published:** 2019-07-03

**Authors:** Carlos Ruiz-Arenas, Alejandro Cáceres, Marcos López-Sánchez, Ignacio Tolosana, Luis Pérez-Jurado, Juan R. González

**Affiliations:** 1 ISGlobal, Centre for Research in Environmental Epidemiology (CREAL), Barcelona, Spain; 2 Universitat Pompeu Fabra (UPF), Barcelona, Spain; 3 CIBER Epidemiología y Salud Pública (CIBERESP), Barcelona, Spain; 4 Genetics Unit, Universitat Pompeu Fabra, Barcelona, Spain; 5 Centro de Investigación Biomédica en Red de Enfermedades Raras (CIBERER), Barcelona, Spain; 6 Hospital del Mar Research Institute (IMIM), Barcelona, Spain; 7 SA Clinical Genetics, Women's and Children's Hospital & University of Adelaide, Adelaide, South Australia Australia; 8 South Australian Health and Medical Research Institute, Adelaide, South Australia Australia; Case Western Reserve University, UNITED STATES

## Abstract

Polymorphic inversions contribute to adaptation and phenotypic variation. However, large multi-centric association studies of inversions remain challenging. We present *scoreInvHap*, a method to genotype inversions from SNP data for genome-wide association studies (GWASs), overcoming important limitations of current methods and outperforming them in accuracy and applicability. *scoreInvHap* calls individual inversion-genotypes from a similarity score to the SNPs of experimentally validated references. It can be used on different sources of SNP data, including those with low SNP coverage such as exome sequencing, and is easily adaptable to genotype new inversions, either in humans or in other species. We present 20 human inversions that can be reliably and easily genotyped with *scoreInvHap* to discover their role in complex human traits, and illustrate a first genome-wide association study of experimentally-validated human inversions. *scoreInvHap* is implemented in R and it is freely available from *Bioconductor*.

## Author summary

Chromosomal inversions are structural variants consisting on an orientation change of a chromosome segment. Inversions have been linked to some phenotypic differences between individuals and to genetic divergence. However, their overall contribution to complex diseases is largely underdetermined as there are no high-throughput methods to call inversion-genotypes in large cohort studies. Here, we propose a new method, *scoreInvHap*, to call individual inversion genotypes from their haplotype similarity. We show that *scoreInvHap* has a high performance when analyzing heterogeneous sources of SNP data. Our current implementation contains 20 human inversions that can be readily genotyped in existing GWAS datasets. We exemplify the utility of *scoreInvHap* by running the first-genome wide association of experimentally validated inversions and a multi-centric inversion association study. All in all, *scoreInvHap* can substantially contribute to increase our knowledge of the role of chromosomal inversions in complex diseases by re-analyzing data from existing genetic association studies.

## Introduction

Frequent polymorphic inversions contribute to adaptation and phenotypic variation [1,2]. However, their global contribution to complex traits remains unknown because there is no specific high-throughput technology to genotype inversions in large cohorts. Previous methods have successfully used SNP data to detect the presence of polymorphic inversions by linkage differences at the breakpoints [3–5] as well as to infer inversion genotypes from the mapping of inversion status to haplotype groups, when the breakpoints are known [6–8]. While inversion calling can be performed by the congruence of different SNP signals [8], only a limited amount of experimentally-validated inversion genotypes have been available for assessing reliable inferences in large cohorts. As such, large association studies that infer inversion genotypes from SNP data have been limited to three human inversions [9–11].

Those inversions that have been successfully genotyped at large scale are either tagged by SNPs (e.g. inv-17q21.31) or their genotypes fully explain the clustering of individuals in the first principal components (PCs) of the SNPs within their breakpoints, such as inv-8p23.1 [6] or inv-16p11.2 [10]. In the later cases, the subject clusters correspond to different haplotype-genotypes (*e*.*g*. A/A, A/B, B/B) of divergent haplotype groups (A and B), supported by the suppression of recombination between inversion states (inverted: I, non-inverted: N) [7]. Few individuals can be then selected for costly experimental genotyping, with methods like FISH, to help labeling the clusters according to the inversion-genotypes (I/I = A/A, I/N = A/B, N/N = B/B). The genotypes of the rest of the subjects are then inferred by haplotype-genotype cluster membership [6]. This unsupervised inference, with posterior experimental labeling of the clusters, has allowed the genotyping of inversions in large cohorts [6–8]. However, this approach is still very limited because individual inferences are based on the analysis of entire population samples, making them computationally inefficient [9] and forcing the reanalysis of the whole dataset when new individuals are included. In addition, it has been observed that some inversions exhibit multiple clusters that exceed the three inversion genotypes and therefore their labeling is unclear [10,12]. Current methods do not address the needs required for the meta-analyses of inversion association studies that include efficient and reliable genotyping in large population samples and inversion-genotype harmonization across different sources of SNP data.

To tackle these problems, we developed *scoreInvHap*, a novel inversion-genotype classifier that enables the inclusion of inversions in regular GWASs. *scoreInvHap* compares how similar the SNPs of a new individual are to those in reference haplotype-genotypes, previously linked to reported experimental inversion-genotypes. Our current implementation enables the efficient and reliable genotyping of 20 human inversions in large cohorts. We studied the performance of the method on the inversion calling of inv-8p23.1 and inv-17q21.31 against two other methods (*invClust* and *PFIDO*) in a wide range of data types: whole genome sequencing, four SNP microarray studies and two exome datasets. We also evaluated the performance of *scoreInvHap* in inv-7p11.2 and inv-Xq13.2 [13], showing that *scoreInvHap* can confidently call inversions with multiple haplotypes. We illustrate how *scoreInvHap* can be used to replicate previous associations of inv-8p23.1 and inv-17q21.31 with autism and schizophrenia, and to perform a genome-wide association study of 15 inversions on breast cancer.

## Results

### *scoreInvHap* for 20 human inversions

**Fig 1:**
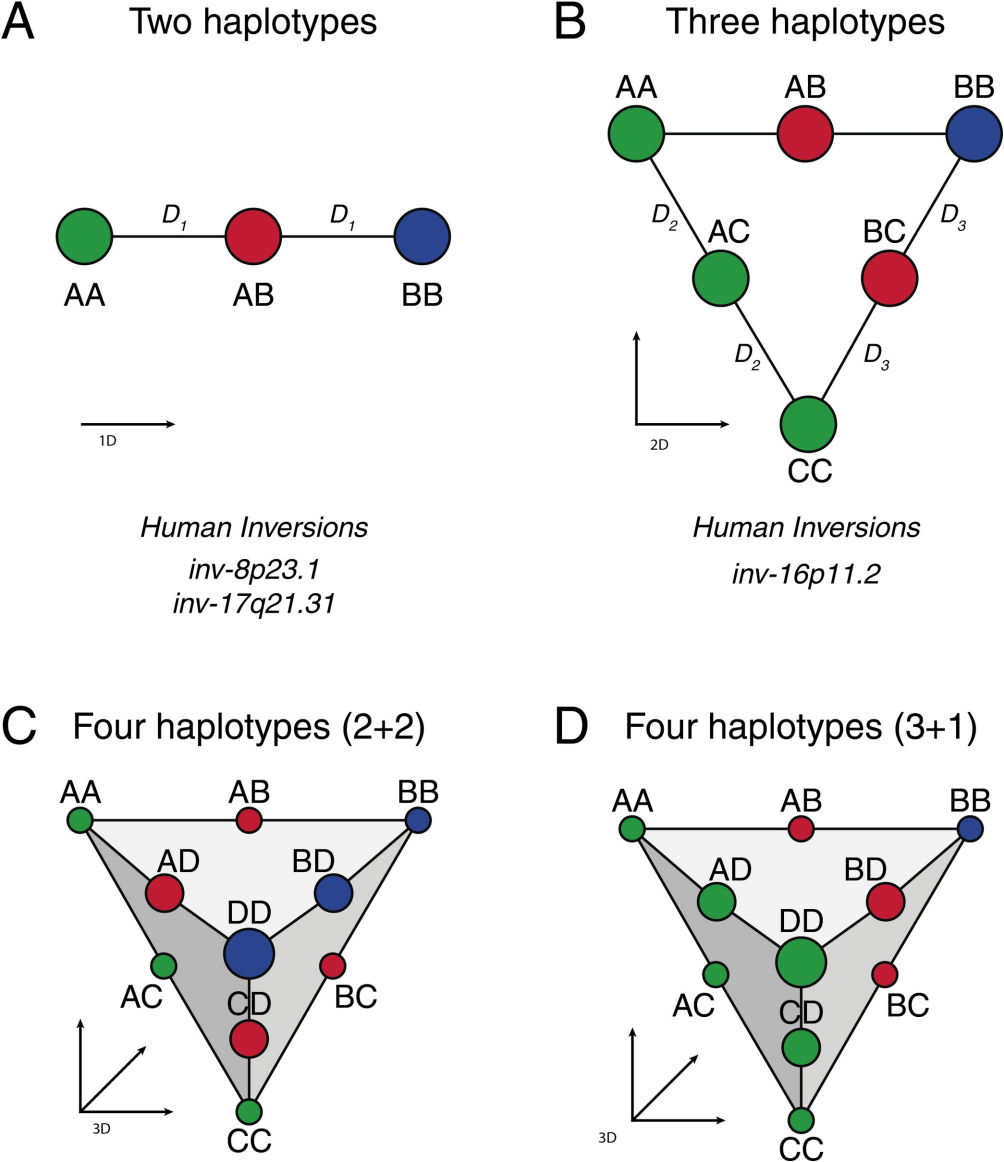
Representation of haplotype-genotype clustering mapped to inversion-genotypes. Disks represent the expected haplotype-genotypes/clusters that can be found in a MDS analysis of SNPs in inverted regions. *Di* are the distances between the clusters that illustrate the equidistance of the heterozygous individuals from the homozygous groups. (A) Inversions supporting two haplotype groups (A and B). Two haplotype groups form three haplotype-genotypes in the first MDS component that map to the inversion-genotypes shown in color (green: standard homozygous, red: heterozygous, blue: inverted homozygous). (B) The first two MDS components show six possible haplotype-genotype groups for three haplotype groups (A, B and C). The homozygous group for the standard allele shows two possible haplotype subpopulations (A and C). (C-D) If a fourth haplotype group (D) is supported by the inversion, the clustering pattern on the first three MDS components should reveal a tetrahedron pattern where the inverted allele can be mapped to either one (1+3), two (2+2) or three (3+1) haplotype groups.

scoreInvHap can generate reliable and scalable inferences for 20 human inversions, whose experimentally validated inversion-genotypes are highly concordant with the haplotype-genotypes of the European individuals of the 1000 Genomes Project [14] (S1 Text, S1 Dataset). These inversions can be genotyped with *scoreInvHap* in any GWAS of European individuals, showing high prediction accuracy on experimental genotypes not used in the classifier (Table 1, S1 Table). Six of these inversions cannot be called with previous methods as they support more than two inversion-haplotypes, revealed by the presence of more than three clusters in the first PCs of the SNPs within the inverted region. We demonstrated that haplotype-inversion labeling for these inversions is recovered at higher PC dimensions, where subject clusters are reliably mapped to numerous haplotype-genotype groups (Fig 1). Using a coalescent simulator for inversions [15], we observed that the existence of more than two haplotype groups is a common feature (S1 Fig).

**Table 1:** 20 human-inversions that can be genotyped from SNP data using *scoreInvHap*.

*scoreInvHap* name	Loc.	Length (Kb)	Inv. Freq.	N haps	Hap-Inv conc. (N)	*scoreInvHap* accuracy	*invClust* accuracy	*PFIDO* accuracy
inv1_004	1p22.1	0.77	11.23	2	99.8**%** (503)	99.8%	99.6%	98.4%
inv1_008	1q31.3	1.2	19.68	2	99.6**%** (503)	99.6%	99.6%	99.0%
inv2_002	2p22.3	0.72	15.11	2	99.8**%** (503)	99.8%	72.2%	72.2%
inv2_013	2q22.1	4.25	71.47	2	100**%** (44)	100%	100.0%	100.0%
inv3_003	3q26.1	2.28	56.16^a^	4	100**%** (38)	100%	62.2%	62.2%
inv6_002	6p21.33	0.87	63.12	2	100**%** (44)	100%	100.0%	100.0%
inv6_006	6q23.1	4.12	6.56	2	99.8**%** (503)	99.8%	87.3%	87.3%
inv7_003	7p14.3	5.25	23.56	2	99.4**%** (503)	99.4%	60.4%	60.2%
inv7_005	7p11.2	73.9	50.39	4	100**%** (28)	100%	50.0%	50.0%
inv7_011	7q11.22	12.7	63.52	2	100**%** (43)	100%	100.0%	97.7%
inv7_014	7q36.1	2.08	19.88	2	99.2**%** (503)	98.4%	98.4%	96.2%
inv8_001	8p23.1	3,925	57.95	2	100**%** (38)	100%	100.0%	100.0%
inv11_001	11p12	4.75	15.81	2	98.8% (503)	97.0%	71.0%	71.0%
inv11_004	11q13.2	1.38	34.39	3	95.8% (503)	95.4%	95.8%	63.6%
inv12_004	12q13.11	19.3	7.46	2	99.8% (503)	99.6%	85.5%	85.5%
inv12_006	12q21.2	1.03	36.98	3	93.4% (503)	93.4%	65.0%	65.0%
inv14_005	14q23.3	0.86	29.42	2	99.6**%** (503)	99.6%	-^c^	56.9%
inv17_007	17q21.31	711	23.96	2	99.5**%** (425)	99.8%	99.8%	97.4%
inv21_005	21q21.3	1.06	51.29	4	99.4**%** (503)	99.4%	99.4%	90.1%
invX_006	Xq13.2	90.8	13.3^b^	4	97.4**%** (38)	97.4%	76.3%	76.3%

Loc.: Citogenetic location. Length: inversion length in kb in hg19. Inv. Freq.: Frequency of the inverted allele in the European individuals of the 1000 Genomes Project Phase 3. N Haps: Number of different haplotypes groups supported by the inversion. Hap-Inv conc.: Percentage of concordance between validated inversion genotypes and haplotype-genotypes clusters in the first PCs of SNPs in the inverted region, showing the level experimental support for inferences based on haplotype to inversion mappings. N: number of individuals with experimental validation of inversion genotypes. a. Inversion genotypes that do not follow Hardy-Weinberg equilibrium. b. inversion frequency was equal for males and females. c. invClust could not genotype this inversion.

### *scoreInvHap* against current methods

We studied the performance of *scoreInvHap* on the inversion calling against *invClust* [8] and *PFIDO* [6]. First, we assessed the methods’ accuracies to predict experimental genotypes in the European subjects of the 1000 Genomes project. We found that *invClust* and *PFIDO* had low accuracy for inversions with more than 2 haplotypes (Table 1) or when the first MDS component did not completely capture the inversion genotype groups (S1 Text).

Second, we tested how sample size affected the calling accuracy of the methods for inversions inv-8p23.1 and inv-17q21.31. We resampled varying number of individuals from 1000 Genomes Project and observed that *scoreInvHap* had high accuracy even when using only one individual reference per haplotype-genotype, whereas *invClust* and *PFIDO* required at least 20 and 30 subjects for accurate classification (S2 Fig). We also run the three methods on the African individuals of 1000 Genome project for inversion 8p23.1 and observed that *scoreInvHap* and *invClust* had lower accuracy (85%), while PFIDO was unable to return a classification. These results can be explained by a low concordance between haplotype-genotypes and experimental inversion-genotypes. Nonetheless, we could not completely rule-out experimental error that penalized the methods’ accuracy (S3 Fig).

**Fig 2:**
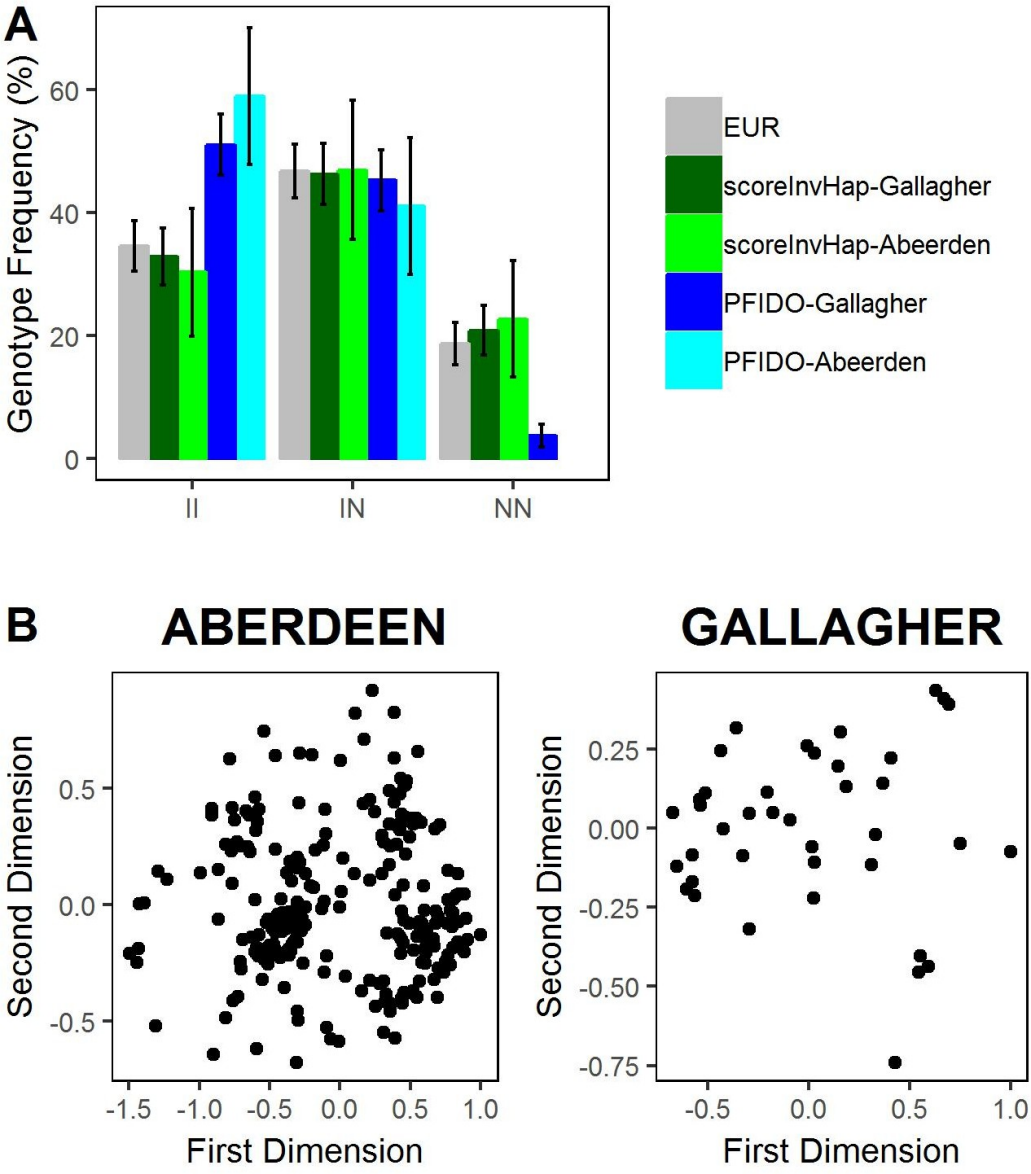
Inversion-genotype frequencies of inv-8p23.1 in exome data as obtained by three genotyping methods. (A) inversion-genotype frequencies for inv-8p23.1 reported by *scoreInvHap* and *PFIDO* in Aberdeen and Gallagher datasets compared with the inversion genotype frequencies of the European individuals in the 1000 Genomes Project. EUR is the frequency in the European individuals of the 1000 Genomes Project. Error bars include the 95% confidence interval of the estimated frequencies. *scoreInvHap*-Gallagher: inversion-genotype frequencies obtained by *scoreInvHap* in the Gallagher dataset. *scoreInvHap*-Aberdeen: inversion-genotype frequencies obtained by *scoreInvHap* in Aberdeen dataset. *PFIDO*-Gallagher: inversion-genotype frequencies obtained by *PFIDO* in Gallagher dataset. *PFIDO*-Aberdeen: inversion-genotype frequencies obtained by *PFIDO* in Aberdeen dataset. (B) First two MDS components of inv-8p23.1 SNPs in Aberdeen and Gallagher datasets showing that *invClust* could not return any genotype classification of the individuals.

Third, we compared the three methods on the inversion calling of inv-8p23.1 and inv-17q21.31 (Table 2, S1 Table) among studies with different sources of SNP data: four SNP microarray studies and two exome sequencing datasets (S2 Table). The four SNP microarray studies came from trio studies, so we could evaluate the transmission errors. Although the three methods returned similar inversion frequencies (S4 and S5 Figs), we observed that *scoreInvHap* and *invClust* had very low transmission errors while *PFIDO* underperformed (Table 2). We did not find any substantial differences among the accuracies of the methods in imputed data (S6 and S7 Figs, Table 2). Inversion calling in the UK10K exome data allowed us to demonstrate the suitability of the method under low SNP coverage. We observed that *scoreInvHap* returned consistent inversion frequencies with those observed for the Europeans of the 1000 Genomes, while *PFIDO*’s frequencies were significantly different and *invClust* failed to identify the inv-8p23.1 genotype clusters (Fig 2, S8 Fig).

**Table 2:** Comparison between scoreInvHap, invClust and PFIDO.

Data type	Measure	Dataset	invClust		PFIDO		scoreInvHap	
			8p23.1	17q21.31	8p23.1	17q21.31	8p23.1	17q21.31
**SNP array data**	Mendelian Errors proportion	AGP	0.1%	0%	-	-	0.2%	0%
		SSC 1Mv1	0.2%	0%	0.2%	2.8%	0.2%	0%
		SSC 1Mv3	0.2%	0%	0.1%	0.3%	0.1%	0%
		SSC Omni	0.2%	0%	0.1%	0%	0.3%	0%
**Imputed SNP array data**	Mendelian Errors proportion	AGP	0.1%	0%	-	-	0.5%	0%
		SSC 1Mv1	0%	0%	0.2%	0.9%	1.1%	0%
		SSC 1Mv3	0.2%	0%	0.4%	0.9%	0.5%	0%
		SSC Omni	0.2%	0%	0.3%	1.3%	0.9%	0%

Finally, we compared the runtime of the three methods on one of the trio datasets (SSC 1Mv3) and found that the parallel version of *scoreInvHap* was the fastest method (S3 Table).

### *scoreInvHap* for inversions with multiple haplotypes

We then demonstrated that the method is efficient in calling genotypes of inversions with multiple haplotype groups. We specifically studied the performance of *scoreInvHap* to call inversion genotypes of inv-7p11.2 and inv-Xq13.2, the largest inversions with multiple haplotype-genotypes (Table 1, S1 Table). We observed that *scoreInvHap* classification matched true inversion-genotypes under low SNP densities (10% of original SNP coverage), for both inversions (S9 Fig). Testing the performance in SNP array data, we observed consistent inversion frequencies with those found for the Europeans of the 1000 Genomes project (S4 Table, S10 and S11 Figs) and found low transmission errors (S5 Table).

### *scoreInvHap* in association studies

**Fig 3:**
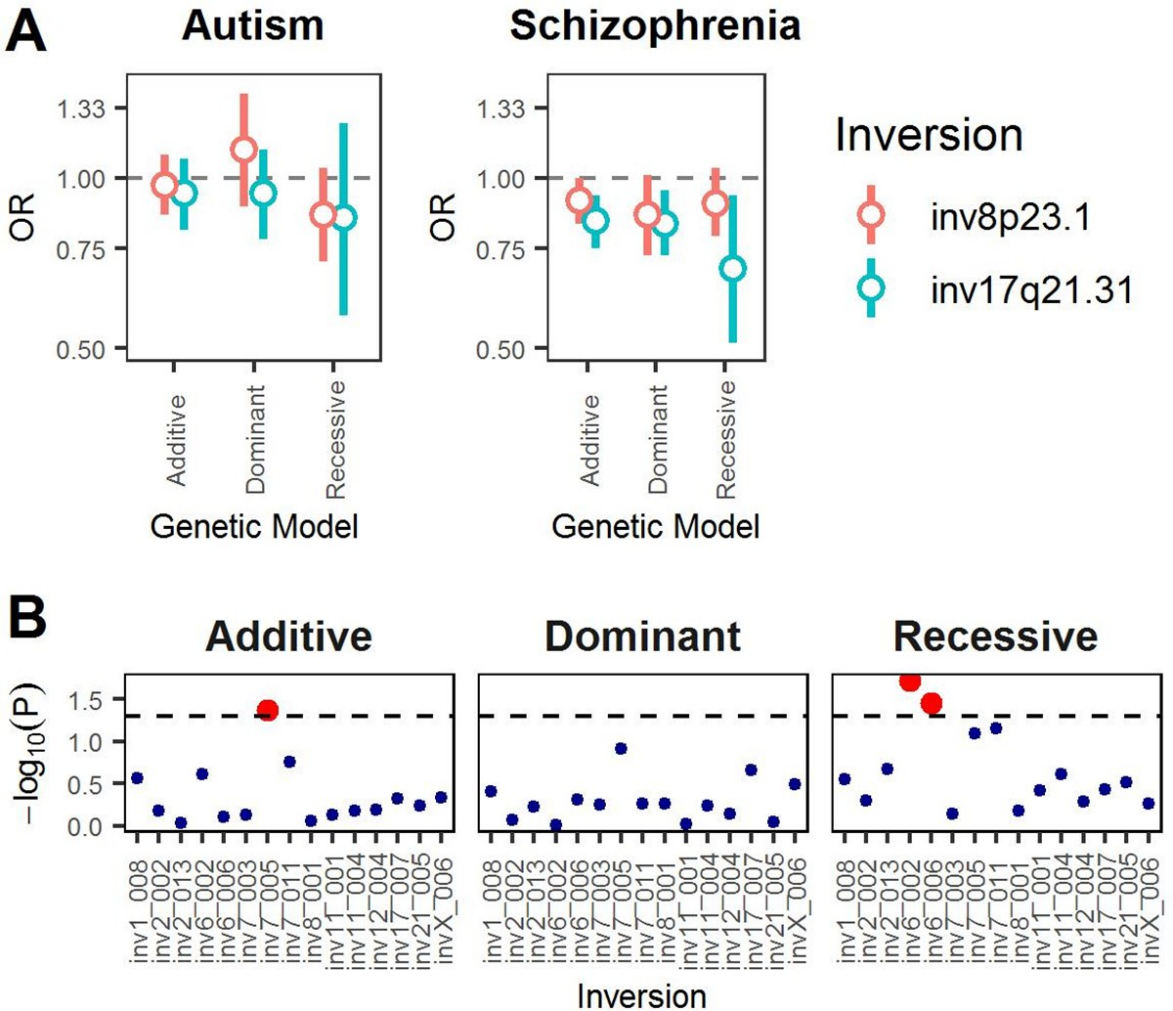
Association studies of inversions genotyped with *scoreInvHap*. (A) Association of inversions inv-8p23.1 and inv-17q21.31 with autism and schizophrenia using exome data in ten studies of UK10K. Horizontal line means no effect. (B) Genome-wide association study of 15 inversions on breast cancer. The horizontal line indicates nominal significance.

We applied *scoreInvHap* to validate initial association analyses of inv-17q21.31 and inv-8p23.1 with autism (cases/controls = 604/5,529) and schizophrenia (cases/controls = 1,308/5,528), using the exome data of UK10K studies [16]. Note that *scoreInvHap* is the only method that allows testing associations with inv-8p23.1 since inversion calling from such a low coverage data could not be performed with other methods (Fig 2). We tested the associations under three inheritance models, adjusting by genome-wide PCs (Fig 3A). We replicated a significant association between schizophrenia and inv-8p23.1 (additive *OR* = 0.91, *P* = 4.9×10^−2^) and inv-17q21.31 (additive *OR* = 0.84, *P* = 1.4×10^−3^). However, we did not replicate the association with autism (Fig 3A) where we could not rule-out remaining differences in genetic ancestry between the studies nor the lack of power for a study with 604 cases and 4358 controls to detect OR~1.12 (power = 0.466), as computed with Genetic Association Study (GAS) Power Calculator [17].

Finally, to illustrate a first genome-wide association study of experimentally-validated human inversions, we tested the association between breast cancer and 15 inversions of Table 1 that could be reliably called in a GWAS study of 1,061 cases and 1,033 controls [18,19]. We did not detect any significant association adjusting for genome-wide PCs, age and multiple comparisons (Fig 3B). However, we did observe associations at a nominal significance level for inversions at 7p11.2 (additive *OR* = 1.14, *P* = 4.2×10^−2^), 6p21.33 (recessive *OR* = 1.36, *P* = 1.8×10^−2^) and 6q23.1 (recessive *OR* = 4.30, *P* = 3.5×10^−2^), which should be further investigated in larger association studies. These applications demonstrate that *scoreInvHap* is a robust genotyping tool of inversions, easy to use on already available GWAS data.

## Discussion

We developed *scoreInvHap*, a new bioinformatics tool to call inversions from SNP data. Its main advantage is the quick call of inversion genotypes from SNP data at the individual level with consistent genotype labeling. As a consequence, inversion genotyping is readily harmonized. Another important advantage is that the method allows the calling of inversion-genotypes using different sets of SNPs. As a result, inversions can be called on datasets with lower SNP coverage than the dataset used for the references as well as to call inversion-genotypes on individuals with missing SNP genotypes.

Previous bioinformatics methods relied on applying a dimensionality reduction technique to SNP data followed by clustering the individuals. Although these methods have been used to associate chromosomal inversions to phenotypic traits [20], they have some limitations. First, these methods partition a population sample into inversion-genotypes but require external information for labeling the inverted-homozygous group, challenging the harmonization of inversion calling in multi-centric studies. Second, the methods are computationally intensive and are inefficient for calling inversion genotypes in large cohorts [9]. Finally, they require a minimum number of individuals to compute accurate calls, so the whole dataset needs to be recalled to include inferences in new individuals. In contrast, the link between haplotypes and inversion status is previous to *scoreInvHap* classification. Consequently, *scoreInvHap* classification is readily comparable across different studies and genotyping techniques (from SNP array to exome data), allowing the harmonization of inversion calling in large meta-analyses. As the method classifies each individual separately, further gains in computational efficiency can be obtained from processing large datasets by batches allowing the genotyping of multiple inversions to be included in association studies.

*scoreInvHap* is the only method designed to genotype inversions with multiple haplotypes, whose abundance in the human genome is likely underestimated. We found inversions with multiple haplotypes on simulations under neutrality and in the inversions reported in *invFEST* and 1000 Genomes. This result suggests that the less common presence of only two haplotypes in inversions inv-8p23.1 and inv-17q21.31 could be due to the reported selection process that occurred in these regions [6,11,21]. Inversions supporting three or four haplotypes have already been described in the literature [10,12]. For inversions inv-7p11.2 and inv-Xq13.2, Aguado and colleagues generated inversion-haplotype trees [13]. Based on the major branches of these trees, they observed that both inversions support four possible haplotype groups, where inv-7p11.2 supports two standard and two inverted haplotypes and inv-Xq13.2 supports three standard and one inverted haplotypes. The tetrahedron structure that we observed for the first three MDS components of these inversions clearly matched the phylogeny of the haplotypes. Sanders and colleagues described more than 100 polymorphic inversions based on a single cell sequencing method [22]. Most of these inversions have not been previously detected with bioinformatics methods designed for inversions with two haplotypes. Therefore, an assessment of the haplotype complexity of these inversions for inference in large association studies is warranted. Further research is also needed for establishing the frequency of complex haplotype patterns in inversions and for elucidating the mechanisms involved in the formation of divergent haplotype groups, supported by the presence of an inversion polymorphism.

*scoreInvHap*, nonetheless, also has limitations. In particular, its performance depends on the representativeness of the reference inversion-genotypes. For inversions inv-8p23.1 and inv-17q21.31 and European samples, we captured the haplotypic variability of the inversions using only one reference per inversion genotype. However, *scoreInvHap* needs to increase the number of experimental references for inversion calling in population samples with higher within haplotype variability, such as inv-8p23.1 in Africans. Further studies are needed to determine the accuracy of the method in inversions with larger genetic variability or populations with admixture.

The inversion genotyping by *scoreInvHap*, like other SNP based methods, is indirect: it does not detect the change of DNA orientation but relies on the haplotype structures generated by inversions. Therefore, it has some clear limitations against experimental methods to detect inversions, such as iPCR [13], next generation sequencing or single strand sequencing [22]. In particular, *scoreInvHap* cannot detect small, recent or de novo inversions, as these inversions do not support different haplotype groups. In addition, *scoreInvHap* will produce wrong classifications for recurrent inversions, where the same haplotype can be found in standard and inverted chromosomes. Despite these limitations, *scoreInvHap* has the advantage of working with stringent conditions of SNP coverage and sample sizes.

All inversions in Table 1 can be genotyped with *scoreInvHap* using SNP data in common formats, like PLINK, snpMatrix or vcf. Performing the genotyping of new inversions in large studies, in human and other species, can be achieved by creating their classifiers within *scoreInvHap*. To build a new classifier, the first step is to demonstrate that a reference sample of individuals can be clustered into haplotype-genotypes. The second step is to show that haplotype-genotypes are unambiguously labeled by experimentally inversion-genotypes. Finally, the reference haplotype-genotypes can be included in the program for genotyping the inversion in new individuals.

We showed how *scoreInvHap* inferences can be used to perform association studies, but additional analyses are needed to understand how the inversion affects the phenotype. One option is that the positional change caused by the inversion affects the regulation of nearby genes, leading to phenotypic differences between individuals. Another option is that the inversion captures the allele (or a combination of alleles) that are the causal variants. Structural variants, such as deletions, copy number alterations or complex re-arrangements, can also be captured by the inversion status and produce the phenotype. Only further analyses can elucidate the mechanism linking an inversion to a phenotype.

In summary, *scoreInvHap* can reliably perform inversion calling for large multi-centric studies with SNP genotype data. The method has been implemented for the calling of 20 human inversions which can be immediately included in any GWAS, to forward our understanding of the role of inversions in complex traits. The method is easily extended to other inversions, in humans and other species, as soon as experimental inversion genotypes become available.

## Materials and methods

### Inversion-haplotype mapping

Inversions suppress recombination within the inverted segment when heterozygous. Therefore, for an ancient non-recurrent inversion, two divergent haplotype groups emerge for each inversion status [7]. Haplotype groups that map to a single inversion status are defined as inversion-haplotypes. In this model, standard and inverted homozygous can be considered as subpopulations where chromosomes belong to the same haplotype group while individuals that are heterozygous for the inversion belong to a 1:1 mixture of standard and inverted chromosomes. This mixture can be seen in the first Multi Dimensional Scaling (MDS) components of the SNPs within the inverted region. In the simplest cases (i.e. inv-17q21.31 and inv-8p23.1), two clear haplotype groups (A and B) emerge for each inversion status (N and I), resulting into three differentiable clusters, or haplotype-genotypes, on the first MDS component (Fig 1A). Heterozygous haplotype-genotype individuals (AB) are visualized equidistant to the homozygous haplotype-genotype groups (AA/BB). Therefore, a univocal map, given by experimental validations, between inversion status and haplotype groups can be established (A = N, B = I). However, in other inversions, more than two haplotype groups have been observed. Inversion at 16p11.2 shows, for instance, a pattern consistent with two haplotype groups (A,C) in the standard configuration and one haplotype group in the inverted allele (B) [10]. In the first MDS components of the SNPs in the region, one can see that heterozygous haplotype-genotype clusters (i.e. AC) are equidistant to their respective homozygous haplotype-genotype clusters (AA and CC), forming a triangular 6-cluster pattern (Fig 1B). Experimental validation is therefore needed to correctly assign an inversion status to each haplotype group (A,C = N, B = I). More complex scenarios are also possible, where four haplotype groups are observed in the region, supporting ten clusters in the first three MDS components consistent to all possible haplotype-genotypes. Experimental inversion-genotypes are then needed to identify the inversion status to which the haplotype-genotypes map.

### Selection of reference haplotype-genotypes

We studied 59 inversions reported in the 503 European individuals of the 1000 Genomes projects. For each inversion, we perform an MDS analysis for all SNPs within the inverted region and studied whether the clustering conformed to a model where haplotype-genotypes could be unambiguously defined. We, therefore, selected the inversions that followed any of the patterns illustrated in Fig 1, increasing the number of MDS components, from 1 to 3, until one of the patterns was clearly identified. This heuristic procedure is described in S1 Text and can be used as a guideline to extend *scoreInvHap* to new inversions. Each cluster of individuals was then identified as a reference group for a given haplotype-genotype to which new individuals are compared for inferring their own haplotype-genotypes. Consequently, at least one reference individual is needed for each haplotype-genotype. The haplotype-genotypes were then mapped to experimental inversion-genotypes to determine their inversion status. At this stage, we measured the degree of concordance between the haplotype and inversion genotypes by their percentage of agreement across individuals, accounting for the cases where more than one haplotype group was found in a single inverted status.

### Algorithm description

We developed *scoreInvHap* for inversions that could be consistently mapped to haplotypes. Therefore, *scoreInvHap* is suitable for those inversions for which the clustering pattern presents no haplotype sharing between the inverted and standard status, and where individuals can be reliably classified into haplotype-genotype groups. We considered that both conditions were fulfilled when clusters followed at least one of the inversion-haplotype mappings in Fig 1.

*scoreInvHap* computes a similarity score between a subject’s SNP genotypes in the inverted region and the haplotype-genotypes that map to experimentally validated inversion-genotypes. Note that the mapping is at the level of SNP and haplotype genotypes and not on individual chromosomes. As such, no phasing is needed for the inferences.

*scoreInvHap* then classifies a new individual into the reference haplotype-genotypes for which their link to inversion-genotypes has been established. The classification is based on similarity scores between the SNP genotypes of the individual and the SNPs in each haplotype-genotype reference group. To compute the score, we first build the classifier from the frequency of each SNP *i* in each reference haplotype-genotype *k* made of *M*_*k*_ reference individuals,

$$f_{ki}(x_{i})=\frac{n_{k}(x)}{M_{k}}$$

where *f*_*ki*_ is the frequency of the *i*-th SNP genotype *x* = {0,1,2} in the haplotype-genotype reference group *k*. The frequency is the ratio between the number of reference individuals (*n*_*k*_) in *k* with SNP genotype *x*_*i*_ and *M*_*k*_. The score of a subject *S*, with *L* (*L* ⊆ *N*) SNP genotypes in the inverted segment (*s*_*1*_,…*s*_*L*_), *s* = {0,1,2}, in the haplotype-genotype reference group *k* is defined as

$$H_{k}=\frac{\sum_{i=1}^{L}f_{ki}(s)\cdot \rho_{i}^{2}}{\sum_{i=1}^{L}\rho_{i}^{2}}$$

where *ρ*_*i*_^*2*^ is the maximum linkage disequilibrium between the SNP *i* and the haplotype groups in the reference individuals. For inversions with two haplotypes, *ρ*_*i*_^*2*^ corresponds to the linkage disequilibrium R^2^ between SNP_*i*_ and the inversion-genotypes. For inversions with three haplotypes (A, B and C), we compute the R^2^ between SNP_*i*_ and each haplotype-genotype. For instance for haplotype A the three haplotype-genotype are given by RR: {BB, BC, CC}, RH: {AB, AC} and HH: {AA}. We use these three haplotype-genotypes to compute the R^2^ between the haplotype group A and SNP_i_ in the reference individuals. *ρ*_*i*_^*2*^ is then, the highest R^2^ across A, B and C.

The inferred haplotype-genotype of the individual *S* is, therefore, the genotype for which the score is maximum, that is a*rg*(*max*{*H*_*1*, …_*H*_*J*_}) where *J* is the total amount of haplotype-genotypes; that is, 3 haplotype-genotypes for 2 haplotype groups, 6 for 3 groups, 10 for 4, and so on (Fig 1). The inversion-genotype for the individual follows from the link between haplotype-genotypes and experimental inversion-genotypes in the reference individuals.

For imputed data, the score is computed as

$$H_{k}=\frac{\sum_{i=1}^{L}\sum_{s_{i}=0,1,2}P_{i}(t)\cdot f_{ki}(s_{i})\cdot \rho_{i}^{2}}{\sum_{i=1}^{S}\rho_{i}^{2}},$$

where *P*_*i*_(*t*) is the probability that the individual *S* has genotype *t*.

### Implementation

We implemented *scoreInvHap* in an R package that supports *snpMatrix* or *VCF* formats, two standard Bioconductor classes for SNP data. The stable version is available in Bioconductor (https://bioconductor.org/packages/release/bioc/html/scoreInvHap.html) while the development version can be installed from the GitHub repository (https://github.com/isglobal-brge/scoreInvHap/). *scoreInvHap* requires the SNP genotypes of an individual in the inversion region. The allele frequencies of SNPs in each genotype reference and the *ρ*^*2*^ between the SNPs and the validated inversion genotypes are built in the classifier and included in the package for the 20 inversions described in Table 1. We have also developed *imputeInversion*, a wrapper to impute SNP array data to use *scoreInvHap*. This tool is available from the GitHub repository (https://github.com/isglobal-brge/imputeInversion).

### Datasets

We used the SNP data (MAF > 5%) of 503 European individuals of 1000 Genomes phase 3 [23]. To test the performance of *scoreInvHap*, *invClust* and *PFIDO* under different conditions, we re-sampled the original dataset under different scenarios. Four scenarios run 200 times each, with different SNPs coverage (10%, 20%, 50% and 75%) for the three methods and six scenarios with different number of individuals (5, 10, 15, 20, 25 and 30) for *invClust* and *PFIDO*. To evaluate *scoreInvHap* performance under different number of individuals, subsets for the references varied from 1 to 5 individuals per haplotype-genotype group. Full *scoreInvHap* performance was tested with a leave-one-out classification approach, classifying one individual with experimental inversion-genotype and using the remaining individuals as references.

We analyzed autism cohorts from the Autism Genome Project (AGP) [24] and the Simon Simplex Collection (SSC) [25]. SSC contained data from three different arrays: Illumina 1Mv1, Illumina 1Mv3 Duo and Illumina HumanOmni 2.5. We considered each array as a different dataset. To include European subjects only, we run a Principal Component Analysis (PCA) using 128 SNP markers for ancestry [26] including all autism cohorts and HapMap3 individuals [27]. We generated a confidence ellipse of 0.99999 around European HapMap subjects and we discarded all individuals outside the ellipse. We discarded 111 subjects of AGP.

We obtained exome data from the UK10K Neurodevelopment datasets. We analyzed two datasets to compare *scoreInvHap* to clustering methods: one of schizophrenia cases (UK10K_NEURO_ABERDEEN) and another of autism cases (UK10K_NEURO_ASD_GALLAGHER). Both datasets are deposited in the European Genome-phenome Archive (EGA) under study accession codes EGAD00001000433 and EGAD00001000436. To select European individuals, we performed a genome-wide PCA of the merge between the UK10K neurodevelopment datasets and two control GWAS datasets: British Birth Cohort (BBC) and National Blood Service (NBD). We discarded subjects outside the central PCA cluster, likewise AGP.

### Inversion simulation

We generated four different inversions using invertFREGENE [15]. We used default values of recombination (1.25×10^−7^) and mutation rates (2.3×10^−7^). In all simulations, the entire simulated region was 2Mb while the inversion comprised 800Kb. Stop frequency was set at 0.4 for the first three inversions and to 0.2 for the forth.

### Inversion genotyping

We run *scoreInvHap* in SNP arrays, imputed data and exome data using the inversion-genotype references included in the package. We discarded SNPs with call rate lower than 0.9. We ran *invClust* using the first two multidimensional scaling components of the SNPs in the inverted regions. We ran *PFIDO* with the default values of SNPs and subject call rate filtering (0.9). We forced the model to return 3 groups and set all the other parameters to default.

### Association analysis

We tested the associations between autism spectrum disorder and schizophrenia, and inversions inv-8p23.1 and inv17q21.31 in ten UK exome studies of the UK10K project (S6 Table). We used subjects from Welcome Trust Case Control Consortium 2 as controls. This dataset consists of two cohorts (National Blood Service (NBS) Cohort and 1,958 British Birth Cohort) genotyped with Illumina 1.2M. We only included individuals classified as Europeans by *peddy* [28]: 5,529 controls, 604 autism cases and 1,308 schizophrenia cases. To run *peddy*, we created two datasets: the first one was the merger between controls and autism cohorts and the other was the merger between controls and schizophrenia. In both cases, we included the 68,689 SNPs that were common between the SNP arrays and the exome data. We applied *scoreInvHap* on each dataset. As cases and controls cohorts belong to different studies, we tested whether the differences in inversion frequencies were not statistically significant (chi-squared test) between the two control cohorts, and among the ten cases cohorts. We used *SNPassoc* for association testing between disease status and inversion genotypes in the joint dataset across all cohorts, adjusting for the joint genome-wide PCs.

We tested the association between inversions and breast cancer on the Cancer Markers of Susceptibility (CGEMS) study [18,19], available in dbGaP (dbGaP Study Accession: phs000147.v3.p1). We only included individuals classified as European with a probability higher than 0.9 inferred by *peddy* [28]: 1,061 cases, 1,033 controls. We imputed the chromosomes containing inversions in Table 1 with Michigan Imputation Server [29]. We selected HRC r1.1 2016 as reference panel and *SHAPEIT* as phasing algorithm. We removed SNPs with an imputation R^2^ smaller than 0.4. We called inversion genotypes with *scoreInvHap* in the 15 inversions having at least 4 SNPs with high quality imputation. We used *SNPassoc* for association testing between disease status and inversion genotypes, adjusting for age and the joint genome-wide PCs.

## Supporting information

S1 Text Generation of *scoreInvHap* references. (PDF)

S1 Dataset Inversion genotypes of the 20 inversions included in *scoreInvHap* for the European individuals of 1000 Genomes. (CSV)

S1 Fig MDS of two different inversions simulated with invertFREGENE. Colors indicate the inversion status of the individuals (green: standard homozygous, red: heterozygous, blue: inverted homozygous). (A) First two MDS components of a simulated inversion showing 6 clusters that map to the inversion genotypes, where standard homozygous support two haplotype groups (case B in Fig 1). (B) First three MDS components of a simulated inversion showing 10 clusters that map to the three inversion-genotypes, where standard homozygous support three haplotype groups (case D in Fig 1).
(PNG)

S2 Fig Accuracy of genotyping inv-8p23.1 and inv-17q21 for three different methods at low samples sizes. (A) Accuracy of *scoreInvHap* vs the number of reference individuals (M_k_) in each haplotype-genotype. We selected inversion references using the same number of individuals for each inversion genotype (i.e. M_NN_ = M_NI_ = M_II_) and computed the accuracy of classifying the other individuals with experimental inversion-genotypes. (B) Accuracy of *invClust* and *PFIDO* vs sample size. Each boxplot is the summary of 200 subsamples without replacement of the same size.
(PNG)

S3 Fig First two MDS components of SNPs in inversion inv-8p23.1 in African individuals of the 1000 Genomes Project. Individuals were colored based on experimental inversion genotypes reported in invFEST. Clusters are clearly differentiated with one standard homozygous close to the heterozygous cluster and one heterozygous individual in the inverted homozygous, suggesting experimental error.
(JPG)

S4 Fig Inversion-genotype frequencies of inv-8p23.1 in the autism cohorts as obtained with the three methods. EUR is the frequency in the European individuals of the 1000 Genomes Project. Error bars include the 95% confidence interval of the estimated frequencies.
(JPG)

S5 Fig Inversion-genotype frequencies of inv-17q21.31 in the autism cohorts obtained with the three methods. EUR is the frequency in the European individuals of the 1000 Genomes Project. Error bars include the 95% confidence interval of the estimated frequencies.
(JPG)

S6 Fig Inversion-genotype frequencies of inv-8p23.1 in the autism cohorts with the three methods with imputed data. EUR is the frequency in the European individuals of the 1000 Genomes Project. Error bars include the 95% confidence interval of the estimated frequencies.
(JPG)

S7 Fig Inversion-genotype frequencies of inv-17q21.31 in the autism cohorts with the three methods with imputed data. EUR is the frequency in the European individuals of the 1000 Genomes Project. Error bars include the 95% confidence interval of the estimated frequencies.
(JPG)

S8 Fig Inversion-genotype frequencies of inv-17q21.31 in exome data as obtained by three genotyping methods. EUR is the frequency in the European individuals of the 1000 Genomes Project. Error bars include the 95% confidence interval of the estimated frequencies. *scoreInvHap*: green, *PFIDO*: blue, *invClust*: red. Dark colors are frequencies in the Aberdeen dataset and light colors are frequencies in the Gallagher dataset.
(JPG)

S9 Fig Accuracy of *scoreInvHap* in 1000 Genomes data for inversions inv-7p11.2 and inv-Xq13.2 and varying SNP coverage. 200 random sets of SNPs were selected at each SNP coverage from the original dataset.
(JPG)

S10 Fig Inversion-genotype frequencies of inv-7p11.2 in the autism cohorts using genotyped and imputed SNP data. EUR is the frequency in the European individuals of the 1000 Genomes Project. Error bars include the 95% confidence interval of the estimated frequencies.
(JPG)

S11 Fig Inversion-genotype frequencies of inv-Xq13.2 in the autism cohorts using genotyped and imputed SNP data. EUR is the frequency in the European individuals of the 1000 Genomes Project. Error bars include the 95% confidence interval of the estimated frequencies.
(JPG)

S1 Table Additional information of human-inversions included in *scoreInvHap*. (DOCX)

S2 Table Datasets used for *scoreInvHap* evaluation. (DOCX)

S3 Table Runtime comparison between the three methods on inv-8p23.1 in SSC 1Mv3 dataset. Table contains the mean and SD runtime in seconds of 10 independent calls.
(DOCX)

S4 Table Summary of inversion population statistics in the autism cohorts. (DOCX)

S5 Table Mendelian Errors in the autism cohorts for inversions inv-7p11.2 and inv-Xq13.2. (DOCX)

S6 Table Datasets used in association analyses of autism and schizophrenia. (DOCX)
