# Supplementary material for: scoreInvHap: Inversion genotyping for genome-wide association studies
Source: PLoS Genet. 2019 Jul 3;15(7):e1008203. doi: 10.1371/journal.pgen.1008203 (PMC6608898; doi:10.1371/journal.pgen.1008203)
Supplement: S1 Text — (PDF) [file pgen.1008203.s001.pdf]

scoreInvHap: inversion genotyping for genome-wide association studies
Supplementary Note

Carlos Ruiz-Arenas, Alejandro Caceres, Marcos Lopez-Sanchez,
Ignacio Tolosana, Luis Perez-Jurado and Juan R. Gonzalez

Contents

1 Selection of 20 inversions **2**
1.1 Inversions supporting two haplotypes 5
1.2 Inversions supporting three haplotypes 8
1.3 Inversions supporting four haplotypes 9
1.4 Other selected inversions 11
1.5 Discarded inversions 12

2 Building *scoreInvHap* references **14**

Generation of *scoreInvHap* references

Experimental inversion genotypes for *scoreInvHap* classification of new individuals were selected for a set of 20 human-inversions. These inversions showed a high concordance between the inversion genotypes of reference individuals and their haplotype-genotypes (Table 1 in manuscript). The SNPs of the reference individuals for each haplotype-genotype were used to create the haplotype classifier based on a similarity score (Methods) between the SNPs of a new subject and the references.

1 Selection of 20 inversions

We initially selected a list of 59 inversions reported in the European individuals of the 1000 Genomes Projects. 42 inversions are given in the 1000 Genomes structural variants database and 21 in the invFEST catalog. Four inversions are common in both sources (Table 1). To check whether haplotypes matched inversion status, we first ran a multidimensional scaling (MDS) of the SNPs inside the inverted regions for all the European subjects of 1000 Genomes project (N=503). We selected inversions with a clear clustering pattern in the first three MDS components, suggesting the presence of differentiated haplotype-genotypes. We then studied whether validated inversion genotypes consistently labeled the clusters. As numerous clusters appeared for some inversions, we developed a strategy to guide the haplotype-inversion mapping. We initially grouped individuals into three plausible haplotype-genotypes, where heterozygous were expected to lie between homozygous groups. When inversions showed more than three clusters, we group individuals in additional clusters to conform with any of the underlying haplotype-genotypes patterns described in Figure 1. In any case, we identified the haplotype-genotypes consistent with the same inversion-genotypes and computed the concordance between inversion and haplotype-genotypes. We then selected inversions with concordance higher than 90%. As a result, we chose 20 human-inversions that can be reliably genotyped from their haplotypes.

Table 1: 59 inversions with experimental inversion-genotypes in the Europeans of the 1000 Genomes Project

Inversion	Original Name	Coordinates	Size (Kb)	Num. SNPs	Selected
inv1_002	INV_delly_INV00003623	chr1:44,059,290-44,059,950	0.66	3	No
inv1_003	CINV_delly_INV00003655	chr1:44,822,001-44,823,172	1.17	2	No
inv1_004	INV_delly_INV00005081	chr1:92,131,841-92,132,615	0.77	6	Yes
inv1_007	HsInv1116	chr1:187,466,478-187,466,727	0.25	0	No
inv1_008	INV_delly_INV00008143 / HsInv0004	chr1:197,756,784-197,757,982	1.20	5	Yes
inv1_009	INV_delly_INV00008409 / HsInv0006	chr1:205,178,526-205,178,807	0.28	3	No
inv1_010	CINV_delly_INV00009421	chr1:240,116,007-240,116,829	0.82	10	No
inv2_001	CINV_delly_INV00055399	chr2:10,825,964-10,827,217	1.25	2	No
inv2_002	CINV_delly_INV00056184	chr2:33,764,554-33,765,272	0.72	6	Yes
inv2_003	CINV_delly_INV00057059	chr2:61,700,635-61,703,542	2.91	4	No
inv2_004	CINV_delly_INV00057450	chr2:72,440,495-72,441,373	0.88	3	No
inv2_010	CINV_delly_INV00060081	chr2:125,051,701-125,053,265	1.56	9	No
inv2_011	CINV_delly_INV00060207	chr2:129,685,049-129,686,125	1.08	3	No
inv2_012	CINV_delly_INV00060877	chr2:131,886,566-131,887,311	0.75	2	No
inv2_013	HsInv0040	chr2:139,004,949-139,009,203	4.25	13	Yes
inv2_014	CINV_delly_INV00062080	chr2:195,979,338-195,984,624	5.29	6	No
inv2_015	HsInv0041	chr2:225,292,980-225,293,082	0.10	0	No
inv3_001	CINV_delly_INV00069534	chr3:7,400,052-7,400,790	0.74	3	No
inv3_002	CINV_delly_INV00072770	chr3:127,496,569-127,497,727	1.16	0	No
inv3_003	HsInv1122	chr3:162,545,362-162,547,641	2.28	6	Yes
inv3_004	CINV_delly_INV00073759	chr3:172,173,323-172,173,704	0.38	0	No
inv4_002	INV_delly_INV00076146	chr4:40,235,048-40,237,039	1.99	7	No
inv5_002	CINV_delly_INV00081296	chr5:31,190,244-31,191,191	0.95	5	No
inv5_003	HsInv0055	chr5:63,764,424-63,775,245	10.82	35	No
inv5_005	CINV_delly_INV00082517	chr5:79,046,344-79,049,460	3.12	7	No
inv5_006	CINV_delly_INV00084159	chr5:147,552,866-147,554,617	1.75	4	No
inv5_007	CINV_delly_INV00084708	chr5:169,597,462-169,598,751	1.29	6	No
inv6_002	HsInv0058	chr6:31,009,222-31,010,095	0.87	5	Yes
inv6_004	HsInv0059	chr6:89,923,634-89,923,942	0.31	2	No
inv6_005	HsInv0061	chr6:107,169,206-107,170,880	1.67	5	No

Table 1 – *Continued from the previous page*

Inversion	Original Name	Coordinates	Size (Kb)	Num. SNPs	Selected
inv6_006	INV_delly_INV00089348	chr6:130,848,198-130,852,318	4.12	12	Yes
inv7_003	CINV_delly_INV00092121	chr7:31,586,765-31,592,019	5.25	11	Yes
inv7_004	INV_delly_INV00092650	chr7:40,879,271-40,880,479	1.21	0	No
inv7_005	HsInv0286	chr7:54,302,450-54,376,389	73.94	180	Yes
inv7_011	HsInv1053	chr7:70,426,185-70,438,879	12.69	10	Yes
inv7_014	CINV_delly_INV00099507	chr7:151,010,030-151,012,107	2.08	5	Yes
inv8_001	HsInv0501	chr8:8,055,789-11,980,649	3924.86	13411	Yes
inv8_002	CINV_delly_INV00102489	chr8:100,157,613-100,158,316	0.70	0	No
inv9_017	HsInv0068	chr9:76,898,140-76,898,389	0.25	0	No
inv9_018	CINV_delly_INV00107146	chr9:107,816,812-107,817,348	0.54	4	No
inv9_019	HsInv1124	chr9:115,873,961-115,875,170	1.21	4	No
inv10_001	INV_delly_INV00010543	chr10:13,104,902-13,105,297	0.40	0	No
inv10_003	INV_delly_INV00012451	chr10:56,767,192-56,773,196	6.00	15	No
inv11_001	CINV_delly_INV00016131	chr11:41,162,296-41,167,044	4.75	7	Yes
inv11_004	CINV_delly_INV00018357	chr11:66,018,563-66,019,946	1.38	5	Yes
inv12_002	INV_delly_INV00021200	chr12:12,544,792-12,546,607	1.82	4	No
inv12_003	CINV_delly_INV00021881	chr12:38,317,979-38,318,554	0.58	0	No
inv12_004	INV_delly_INV00022081	chr12:47,290,470-47,309,756	19.29	43	Yes
inv12_005	CINV_delly_INV00023069	chr12:70,680,610-70,682,061	1.45	2	No
inv12_006	CINV_delly_INV00023088	chr12:71,532,784-71,533,816	1.03	4	Yes
inv12_007	CINV_delly_INV00023095	chr12:71,709,550-71,710,286	0.74	0	No
inv14_005	CINV_delly_INV00029966	chr14:65,842,304-65,843,165	0.86	4	Yes
inv14_006	CINV_delly_INV00029989	chr14:67,170,328-67,171,706	1.38	0	No
inv16_016	HsInv0030	chr16:75,240,199-75,256,700	16.50	95	No
inv16_017	INV_delly_INV00040664 / HsInv0031	chr16:85,188,639-85,189,823	1.18	9	Yes
inv17_017	HsInv0573	chr17:43,661,775-44,372,665	710.89	3637	Yes
inv21_005	INV_delly_INV00066155 / HsInv0045	chr21:28,020,653-28,021,711	1.06	11	Yes
invX_002	HsInv0072	chrX:45,548,349-45,550,615	2.27	5	No
invX_006	HsInv0396	chrX:72,215,927-72,306,774	90.85	135	Yes

1.1 Inversions supporting two haplotypes

In some inversions, we clearly differentiated two haplotype groups (A, B), each of which maps to an inversion state (A=I, B=N). The haplotype groups were detected from the clustering of the first components of a MDS of the SNPs in the inversion. In these cases, three equidistant clusters were observed, corresponding to three haplotype-genotypes (A/A, A/B, B/B) that were univocally identified with the inversion-genotypes (I/I, I/N, N/N). The heterozygous cluster was always found between the homozygous clusters. We observed that 14 inversions could be included in this group.

We observed that four (inv1_008, inv8_001, inv14_005 and inv17_007) of the 14 inversions showed three clusters only (Figure 1). Individuals with experimentally genotyped inversions were then used to label the haplotype-genotype groups, showing a high concordance between haplotype and inversion status; see colored individuals and histograms in Figure 1.

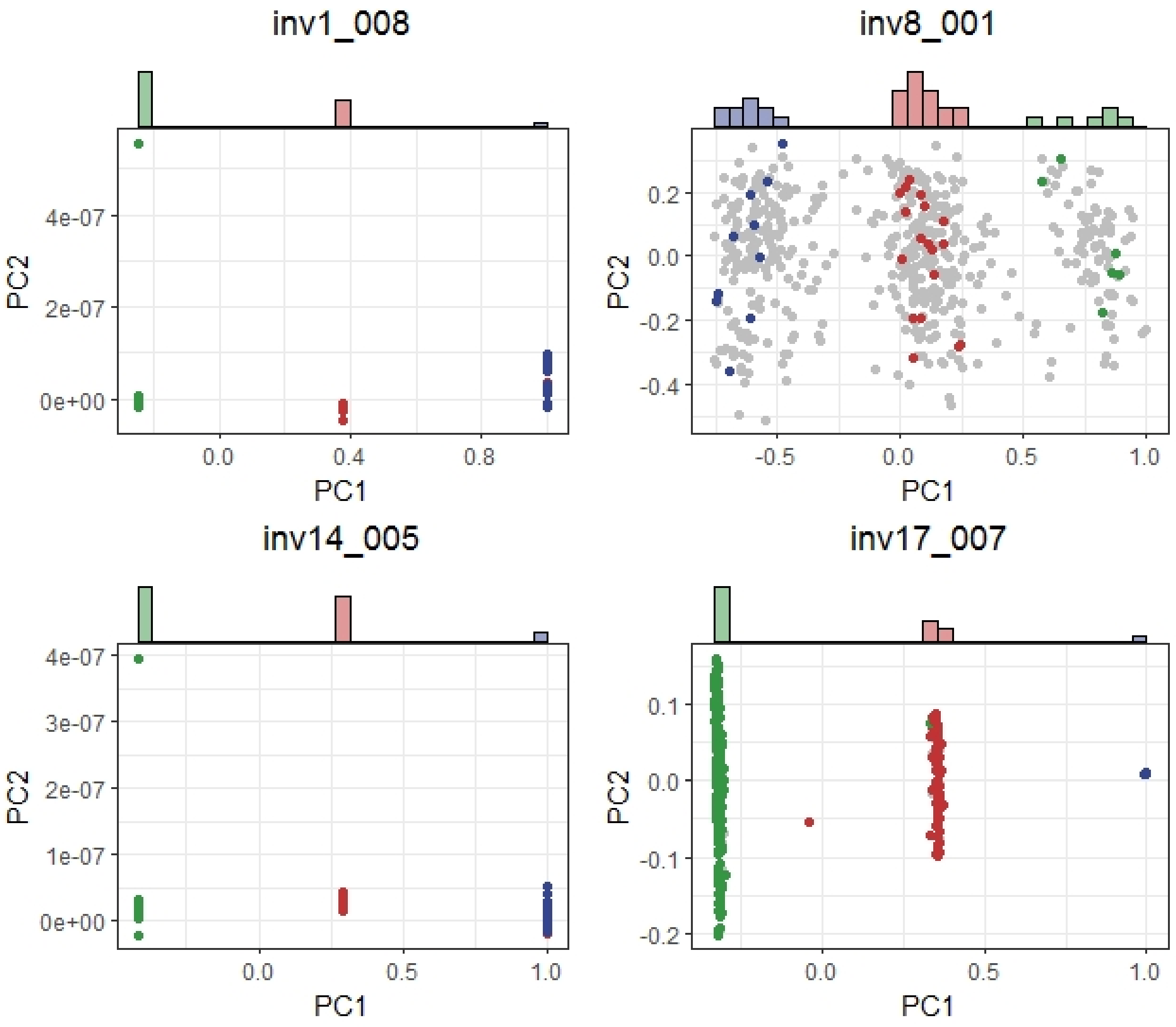

Figure 1: First MDS components of SNPs in inversions supporting two haplotypes. The figures show three clusters that distinguish different haplotype-genotype groups. Individuals are colored depending on their experimental inversion genotypes (green: homozygous standard, red: heterozygous, blue: homozygous inverted, grey: unknown)

We observed that for five other inversions (inv1_004, inv2_013, inv6_002, inv7_011, inv7_014),

although there was within cluster variability in the first MDS components, the clustering separated well the inversion-genotypes (Figure 2).

We observed two inversions (inv2_002 and inv6_006) where clear clusters could be reliably labeled by inversion-genotype but using the second and third MDS components (Figure 3).

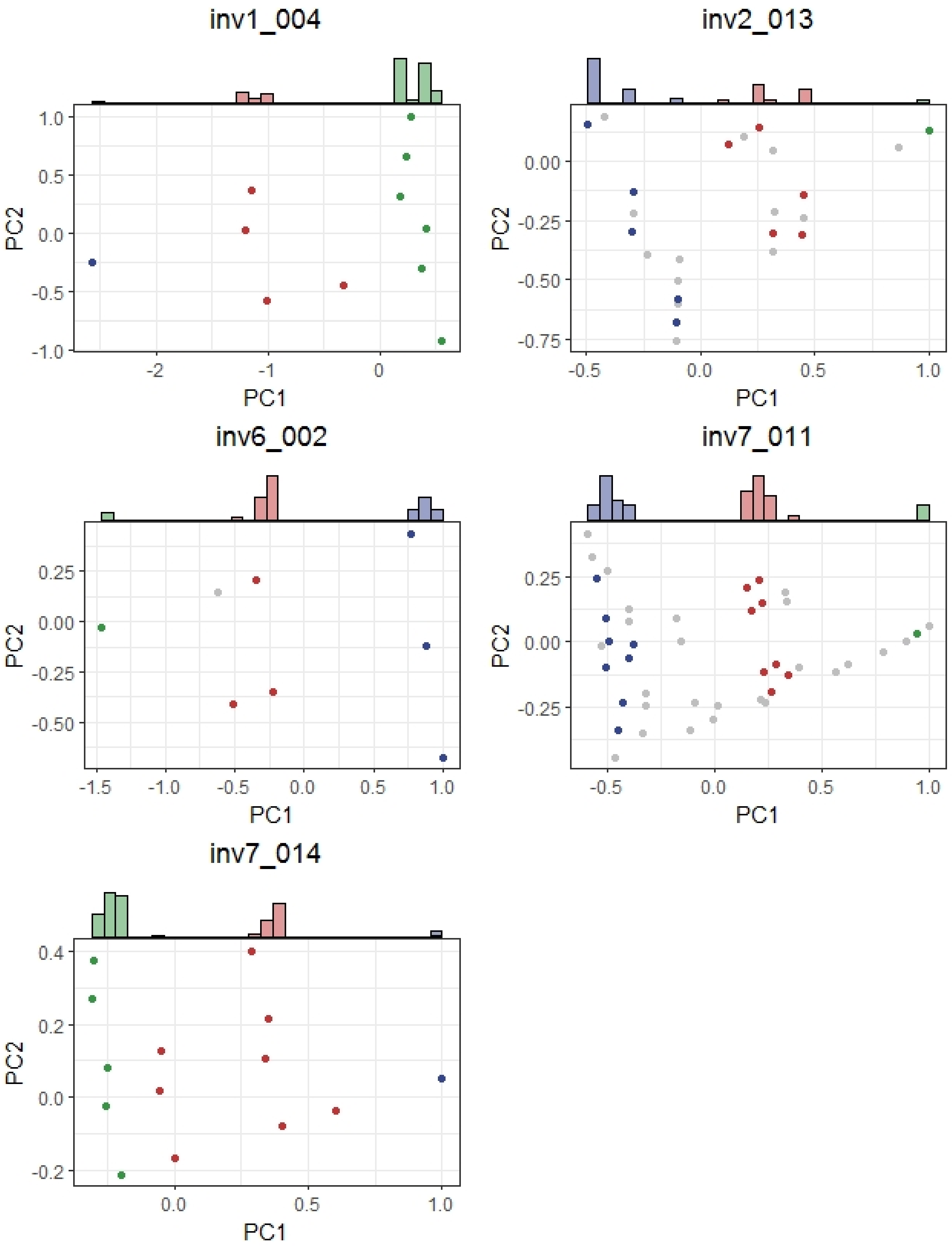

Figure 2: First MDS components of SNPs in inversions supporting two haplotypes. The figure shows three clusters that distinguish different haplotype-genotype groups but with increased within-cluster variability. Individuals are colored depending on their experimental inversion genotypes (green: homozygous standard, red: heterozygous, blue: homozygous inverted, grey: unknown)

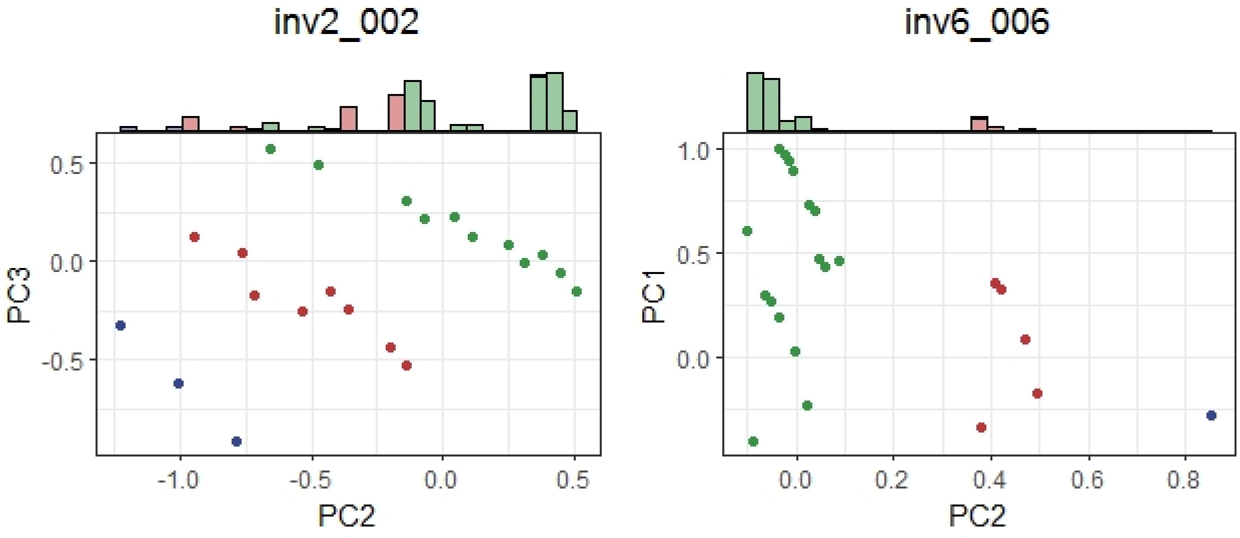

Figure 3: MDS components of SNPs in inversions supporting two haplotypes. The figure shows three clusters that distinguish different haplotype-genotype groups in other MDS components than the first one. Individuals are colored depending on their experimental inversion genotypes (green: homozygous standard, red: heterozygous, blue: homozygous inverted, grey: unknown)

1.2 Inversions supporting three haplotypes

In other inversions, we clearly differentiated three haplotype groups (A, B, C), two of which mapped to the same inversion state (e.g. A=I, B=N, C=N). For these inversions, the first MDS components showed 6 haplotype-genotype clusters, distributed in a triangular shape in which the clusters at the vertices correspond to haplotype-homozygous (A/A, B/B, C/C) and haplotype heterozygous (A/B, B/C, A/C) are in the edges equidistant to their homozygous groups. Experimental inversion-genotypes are used to label the haplotype groups that belong to the same inversion status.

We observed two inversions (inv11_004 and inv12_006) that supported three haplotypes (Figure 4). In inv11_004, the first component discriminated clusters by inversion-genotype while for inv12_006 we needed the two first components. Both inversions had few individuals some distance outside the expected haplotype-genotype cluster. We defined 6 haplotype-genotypes reference groups, each of which is highly concordant to a given experimental inversion-genotype.

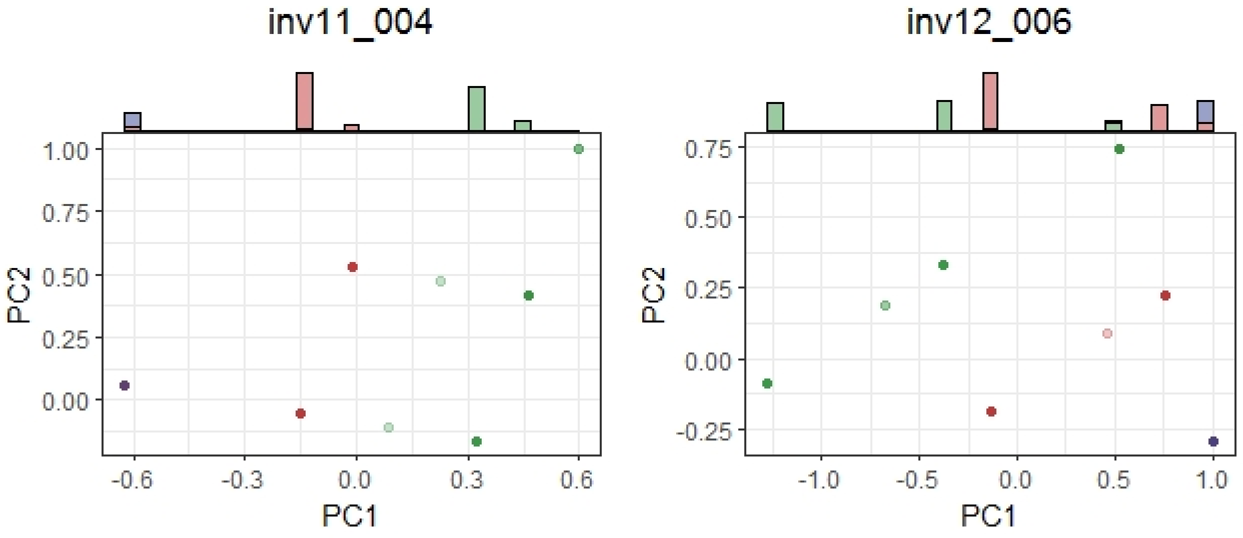

Figure 4: MDS of inversions with three haplotypes. Individuals are colored depending on their validated inversion genotypes (green: homozygous standard, red: heterozygous, blue: homozygous inverted, grey: unknown). Pale clusters have few samples.

1.3 Inversions supporting four haplotypes

In these inversions, we clearly differentiated four haplotype groups (A, B, C, D), which could be mapped to any inversion state (e.g. A=I, B=N, C=N, D=I). This clustering pattern observed for these inversions is an extension of the three haplotypes triangle to an additional dimension. Thus, clusters at vertices correspond to haplotype-homozygous individuals while haplotype-heterozygous individuals are on the edges between them.

We found four inversions (inv3_003, inv7_005, inv21_005 and invX_006) that clearly supported four haplotype groups (Figure 5), visualized in the first three MDS components. For these inversions, we made 10 clusters, corresponding to the number of possible haplotype-genotypes, and we then confirmed that the clusters were concordant to the experimental inversion genotypes.

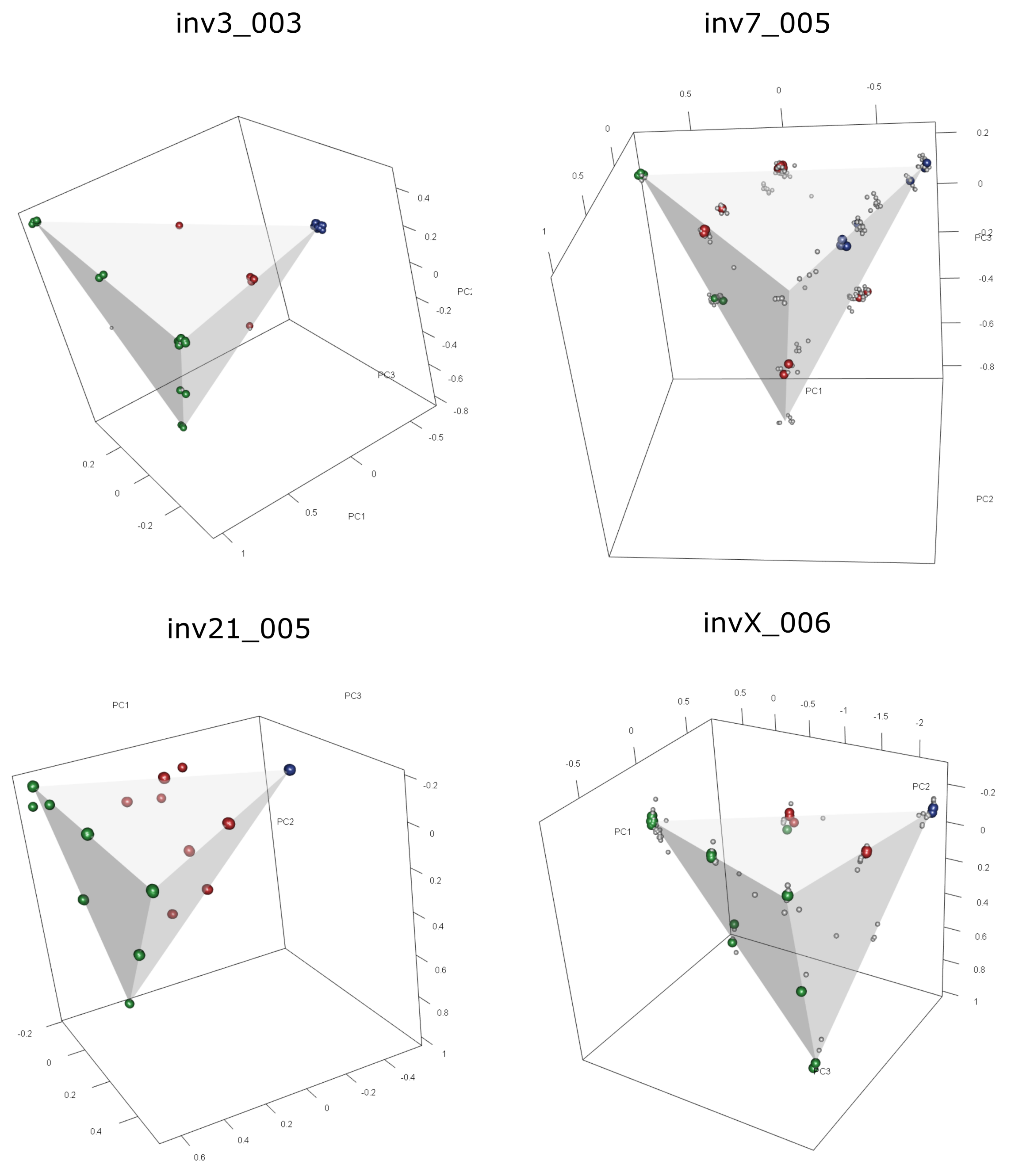

Figure 5: MDS of inversions with four haplotypes. Individuals are colored depending on their validated inversion genotypes (green: homozygous standard, red: heterozygous, blue: homozygous inverted, grey: unknown)

1.4 Other selected inversions

We selected three additional inversions (inv7_003, inv11_001 and inv12_004) that although they showed large variability within the inversion-genotype groups, the haplotype-genotypes could be reliably labeled by them (Figure 6). In inversions inv11_001 and inv12_004, the second component separated individuals by inversion-genotype, while for inv7_003 the second and the third components were needed. For these inversions, two haplotype groups could be differentiated and therefore three haplotype-genotypes references were created.

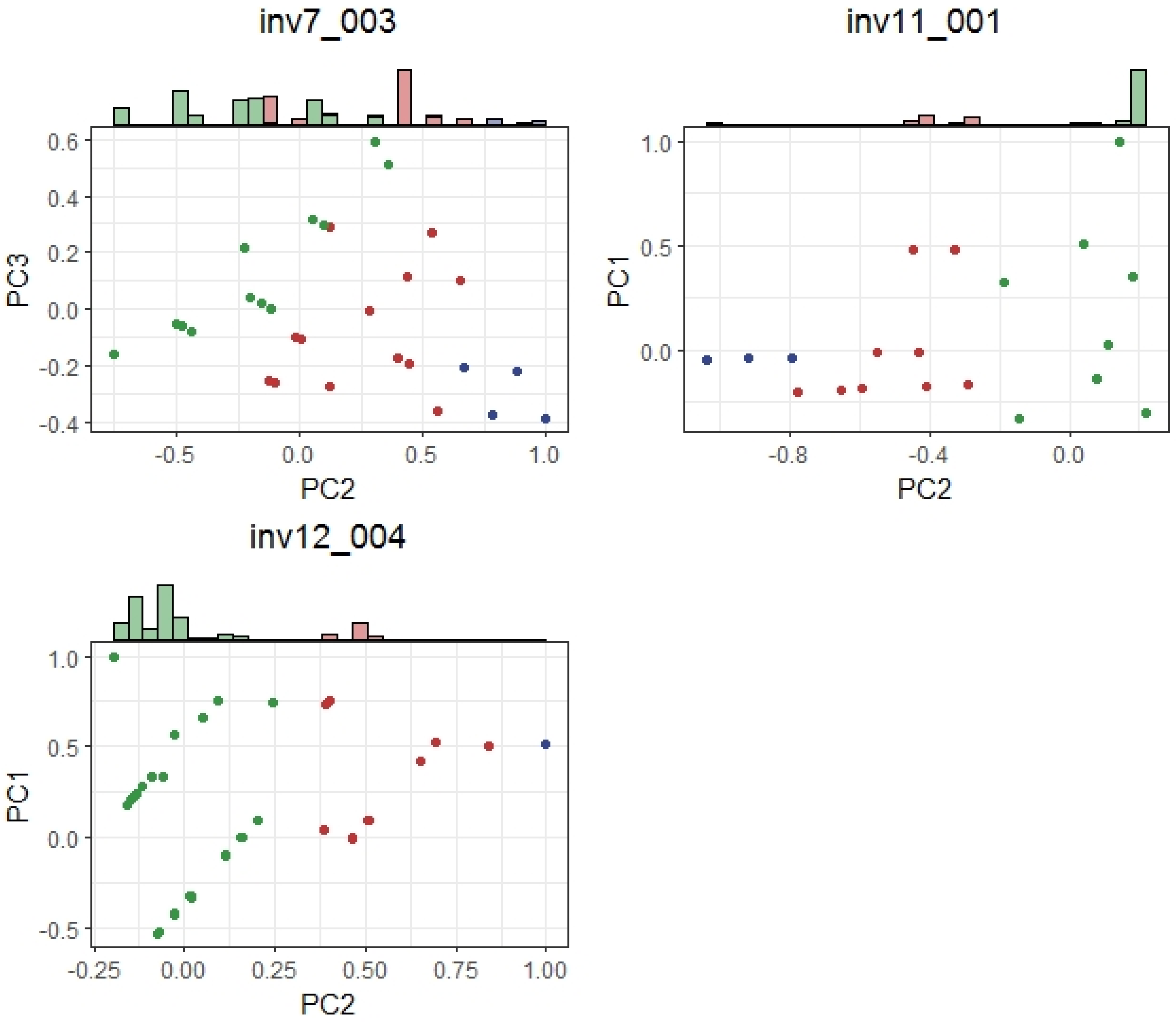

Figure 6: MDS of inversions with four haplotypes. Samples are colored depending on their validated inversion genotypes (green: homozygous standard, red: heterozygous, blue: homozygous inverted, grey: unknown)

1.5 Discarded inversions

We observed one inversion with three clear haplotype-genotypes although the concordance between inversion-genotypes and haplotype-genotypes was 86% (Figure 7). Most discrepancies were for inverted-heterozygous found in the haplotype-homozygous group. In addition, we observed that inversion genotypes from 1000 Genomes were not in Hardy-Weinberg equilibrium (p-value < 3.95^{-5}), with an over-enrichment of heterozygous samples. This suggests that the low concordance could be due to an experimental error in genotyping heterozygous samples. Further experimental data is then required.

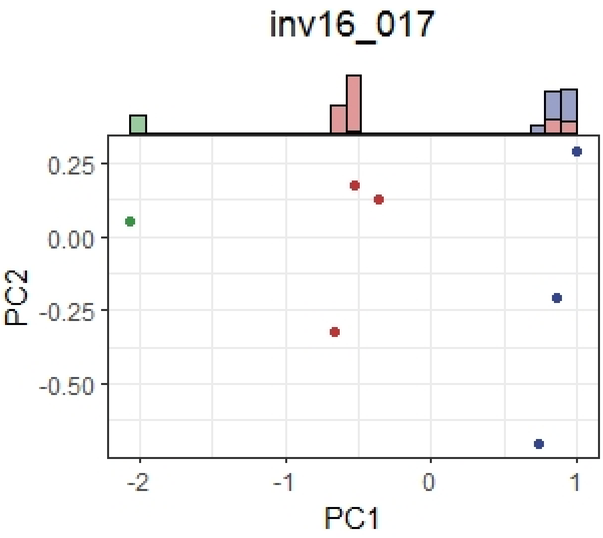

Figure 7: MDS of inv16_017. Individuals are colored depending on their validated inversion genotypes (green: homozygous standard, red: heterozygous, blue: homozygous inverted, grey: unknown)

Finally, we illustrate some examples of inversions that we discarded (Figure 8). Some inversions were discarded because the MDS generated structures incompatible with the presence of expected haplotype-genotypes groups (inv16_016). In other cases, the labeling of haplotype-genotypes with inversion-genotypes was not consistent (inv2_010 and inv10_003). We also discarded inversions that did not contain SNPs with a MAF > 0.05 in the European individuals of 1000 Genomes Project.

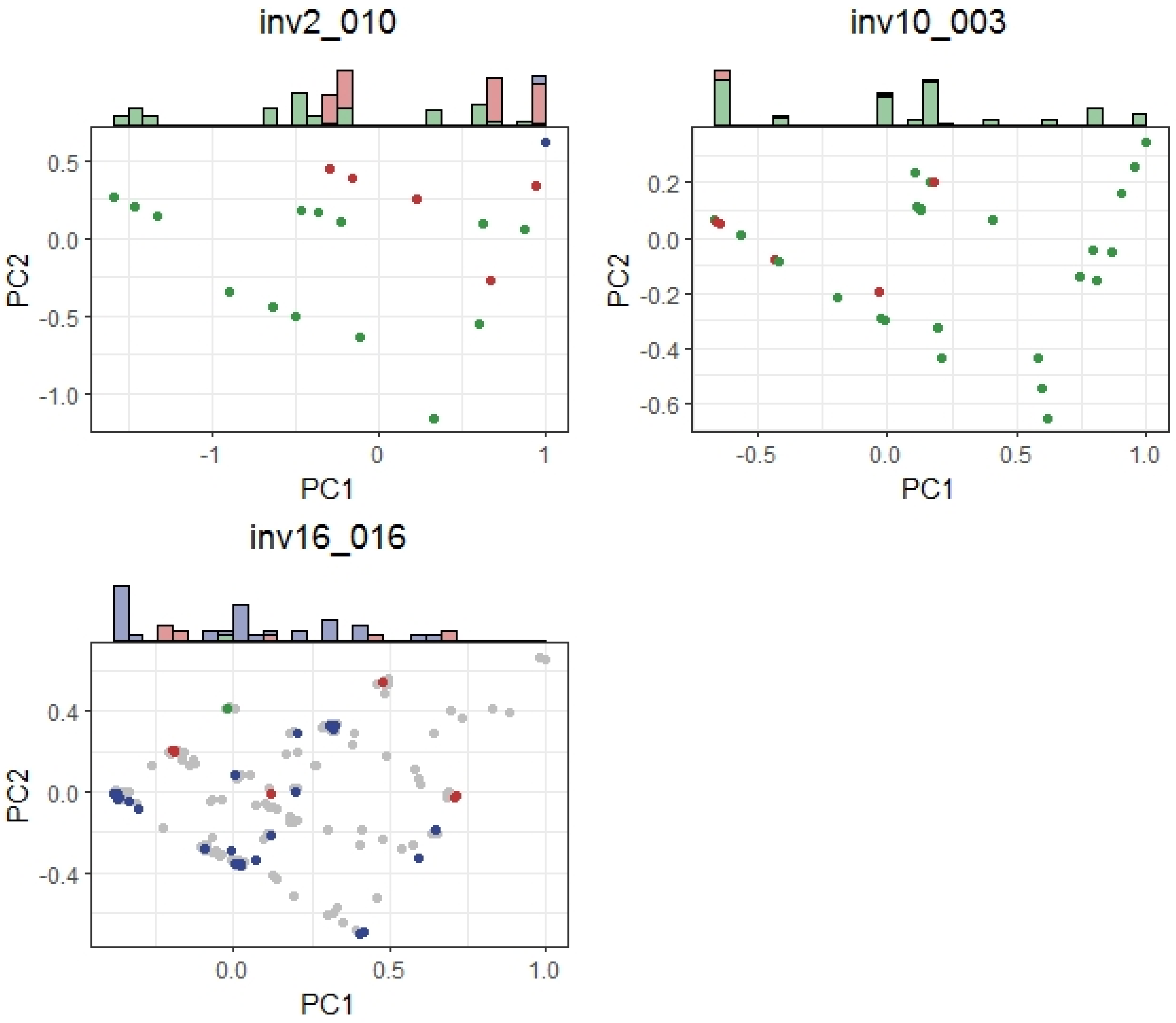

Figure 8: MDS of discarded inversions. Individuals are colored depending on their validated inversion genotypes (green: homozygous standard, red: heterozygous, blue: homozygous inverted, grey: unknown)

2 Building *scoreInvHap* references

scoreInvHap references were made from all European individuals of the 1000 genomes project. Subjects were classified into an haplotype-genotype as explained before. Each haplotype-genotype corresponds to an inversion-genotype experimentally labelled. Haplotypes (a, b) with the same inversion status are noted with lower case letters (e.g. Na, Nb or Ia, Ib).

The *scoreInvHap* classifier is built on four sources of information: I) the genotype frequencies of each SNP in each of the reference haplotype-genotypes, II) the maximum linkage between the SNPs and all haplotype-genotypes, III) SNP alleles in 1000 Genomes to map SNPs from other datasets to *scoreInvHap* references and IV) the correspondence between the haplotype-genotypes and some experimental inversion-genotypes. All the information is based on the 503 European individuals of 1000 Genomes Project.
