# Supplementary figures and images for: scoreInvHap: Inversion genotyping for genome-wide association studies
Source: PLoS Genet. 2019 Jul 3;15(7):e1008203. doi: 10.1371/journal.pgen.1008203 (PMC6608898; doi:10.1371/journal.pgen.1008203)

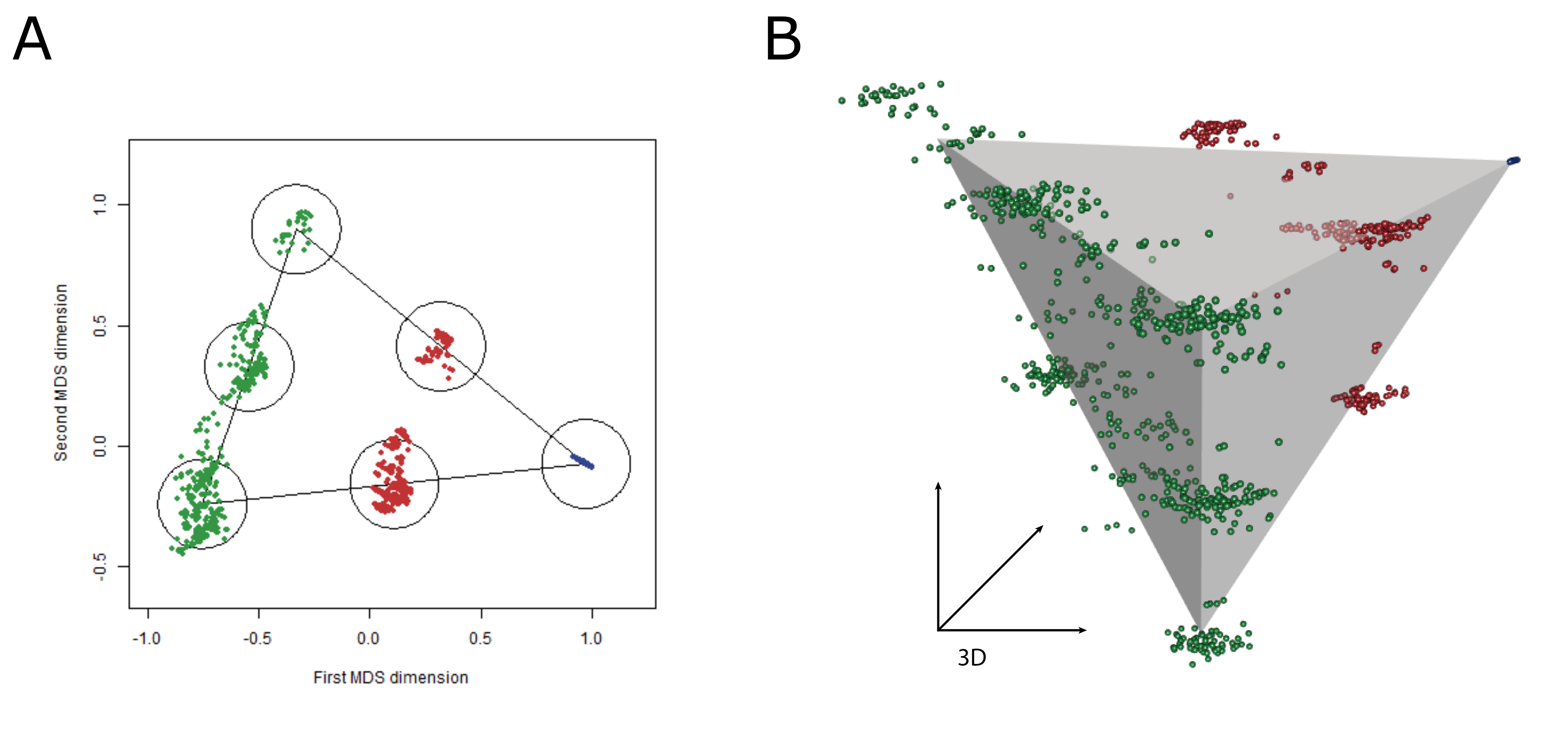

Supplement: S1 Fig — Colors indicate the inversion status of the individuals (green: standard homozygous, red: heterozygous, blue: inverted homozygous). (A) First two MDS components of a simulated inversion showing 6 clusters that map to the inversion genotypes, where standard homozygous support two haplotype groups (case B in Fig 1). (B) First three MDS components of a simulated inversion showing 10 clusters that map to the three inversion-genotypes, where standard homozygous support three haplotype groups (case D in Fig 1). (PNG) [file pgen.1008203.s003.png]

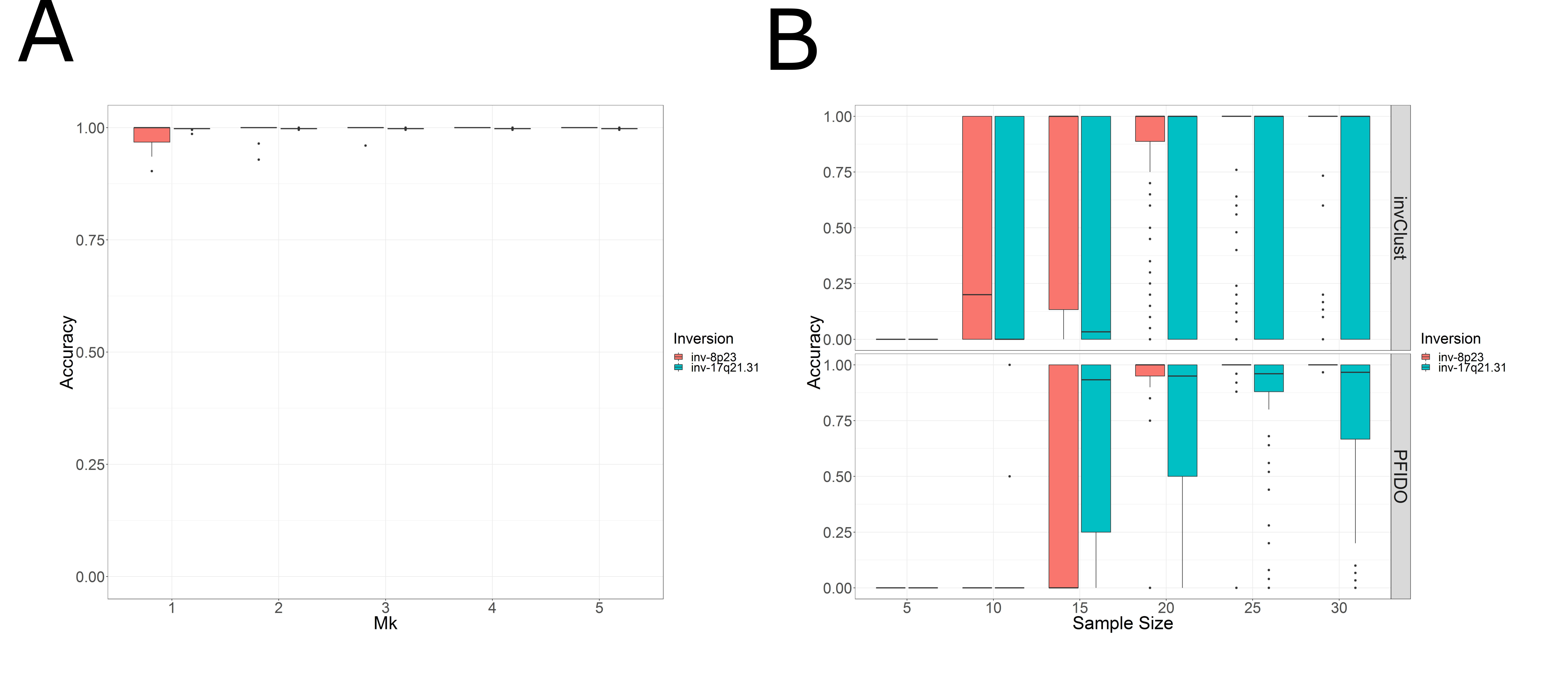

Supplement: S2 Fig — (A) Accuracy of scoreInvHap vs the number of reference individuals (Mk) in each haplotype-genotype. We selected inversion references using the same number of individuals for each inversion genotype (i.e. MNN = MNI = MII) and computed the accuracy of classifying the other individuals with experimental inversion-genotypes. (B) Accuracy of invClust and PFIDO vs sample size. Each boxplot is the summary of 200 subsamples without replacement of the same size. (PNG) [file pgen.1008203.s004.png]

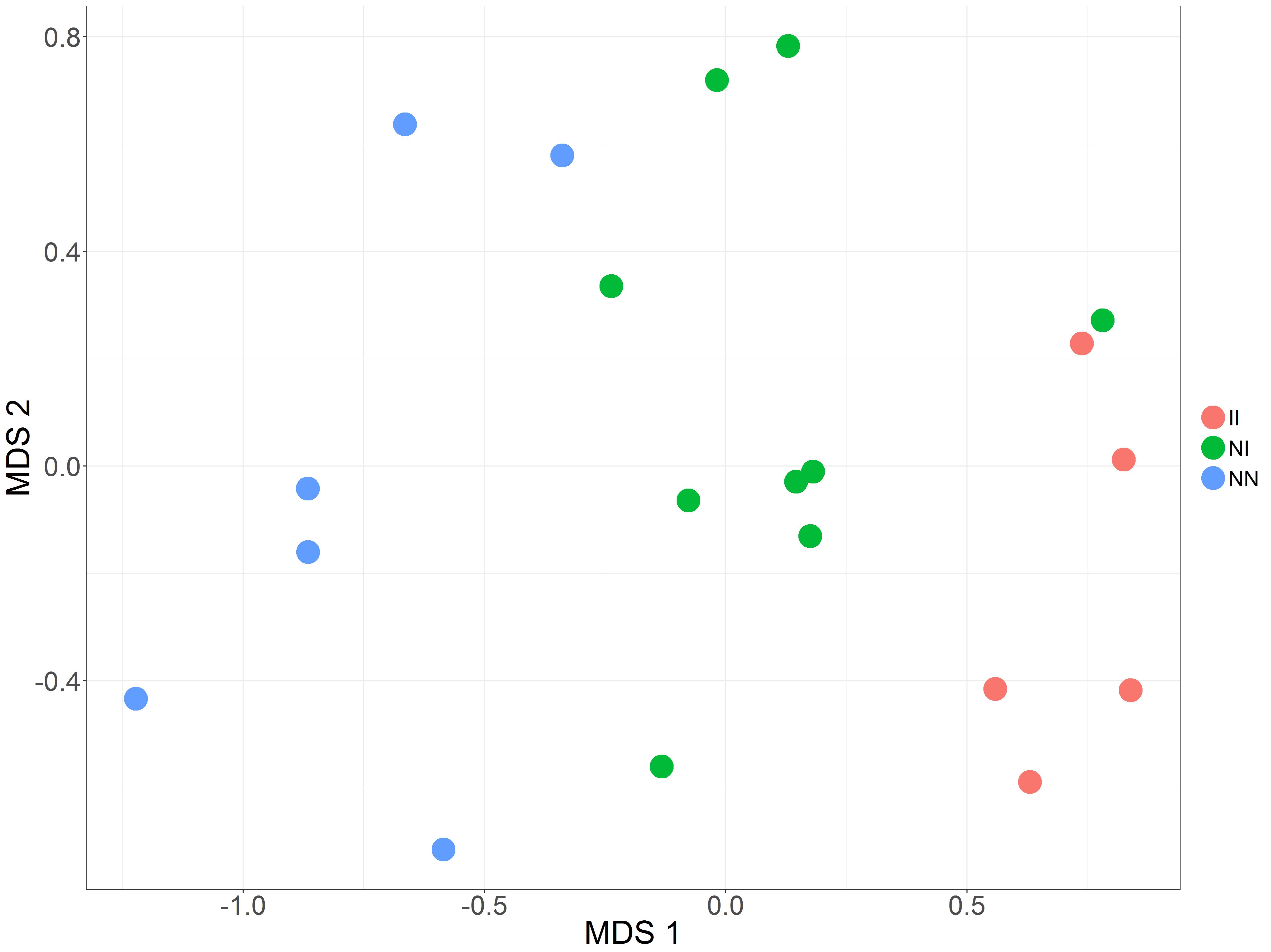

Supplement: S3 Fig — Individuals were colored based on experimental inversion genotypes reported in invFEST. Clusters are clearly differentiated with one standard homozygous close to the heterozygous cluster and one heterozygous individual in the inverted homozygous, suggesting experimental error. (JPG) [file pgen.1008203.s005.jpg]

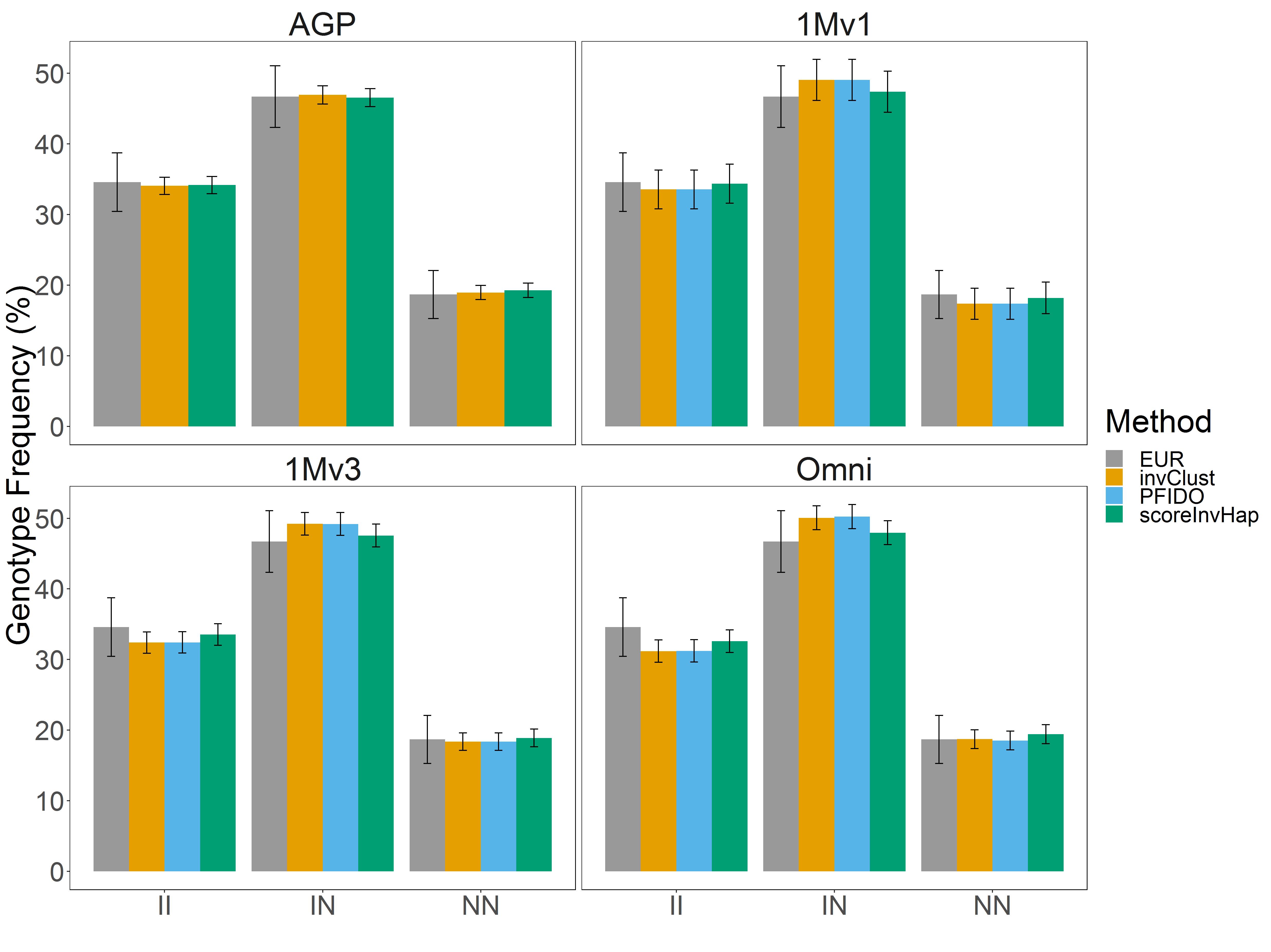

Supplement: S4 Fig — EUR is the frequency in the European individuals of the 1000 Genomes Project. Error bars include the 95% confidence interval of the estimated frequencies. (JPG) [file pgen.1008203.s006.jpg]

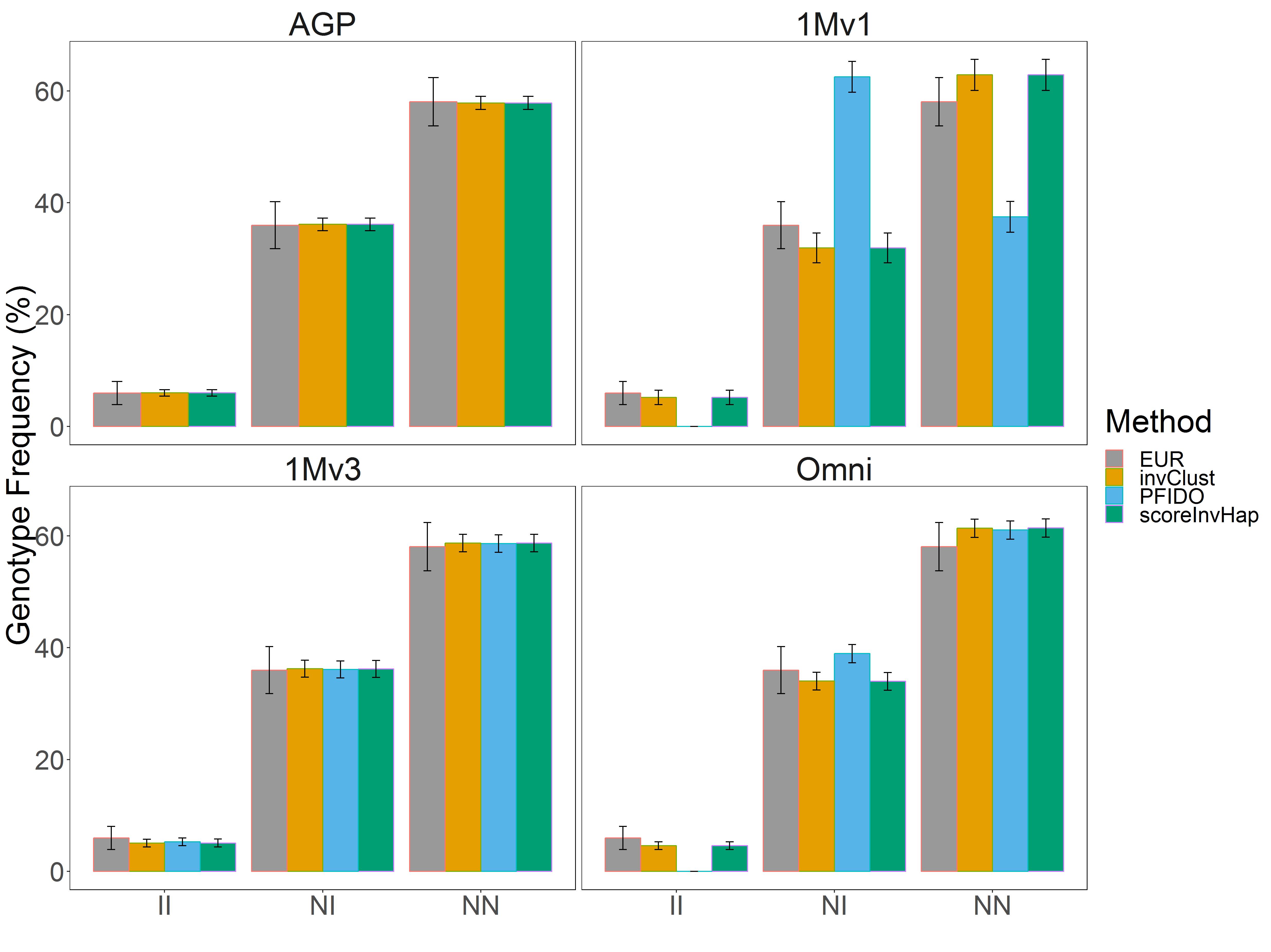

Supplement: S5 Fig — EUR is the frequency in the European individuals of the 1000 Genomes Project. Error bars include the 95% confidence interval of the estimated frequencies. (JPG) [file pgen.1008203.s007.jpg]

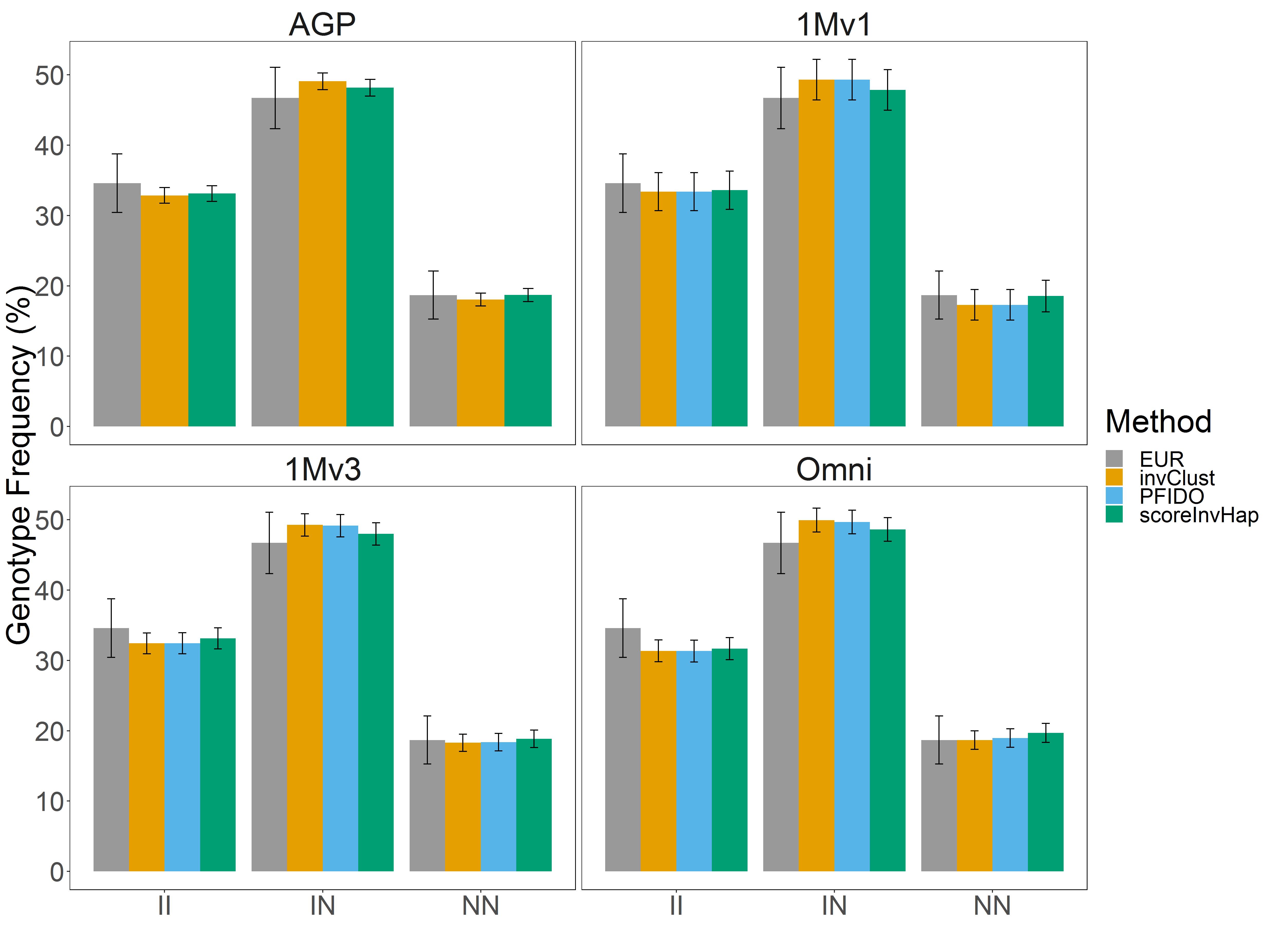

Supplement: S6 Fig — EUR is the frequency in the European individuals of the 1000 Genomes Project. Error bars include the 95% confidence interval of the estimated frequencies. (JPG) [file pgen.1008203.s008.jpg]

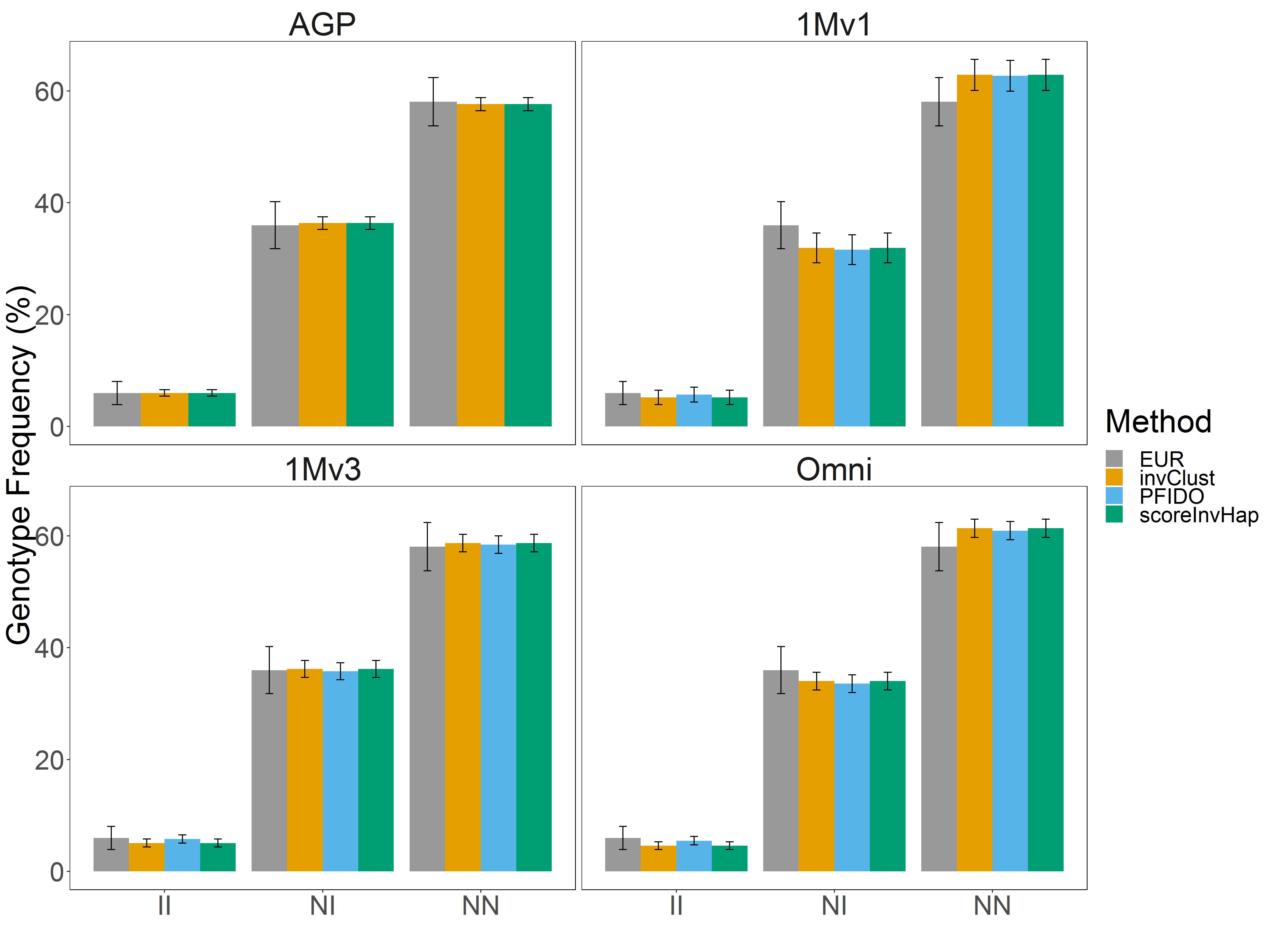

Supplement: S7 Fig — EUR is the frequency in the European individuals of the 1000 Genomes Project. Error bars include the 95% confidence interval of the estimated frequencies. (JPG) [file pgen.1008203.s009.jpg]

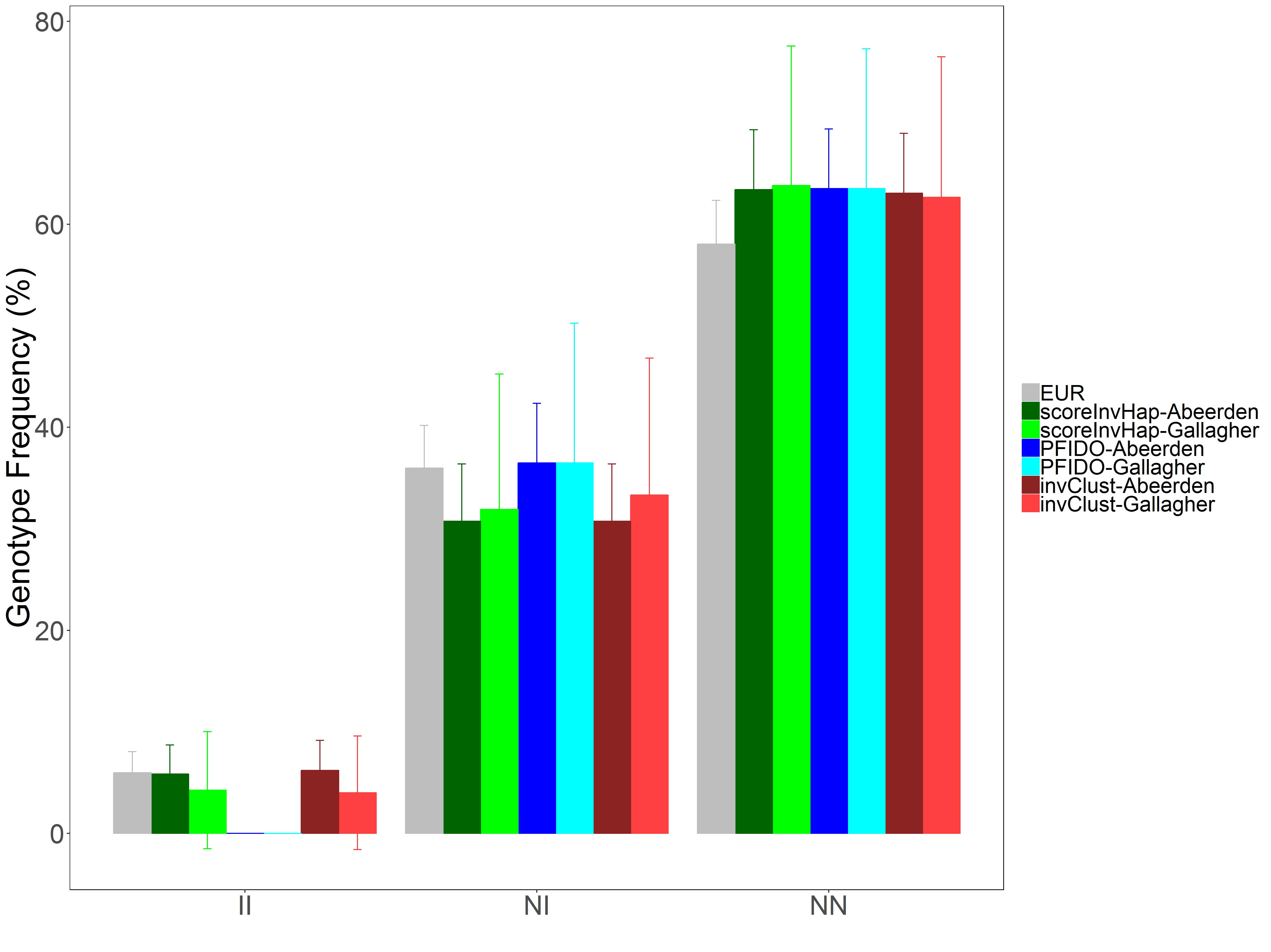

Supplement: S8 Fig — EUR is the frequency in the European individuals of the 1000 Genomes Project. Error bars include the 95% confidence interval of the estimated frequencies. scoreInvHap: green, PFIDO: blue, invClust: red. Dark colors are frequencies in the Aberdeen dataset and light colors are frequencies in the Gallagher dataset. (JPG) [file pgen.1008203.s010.jpg]

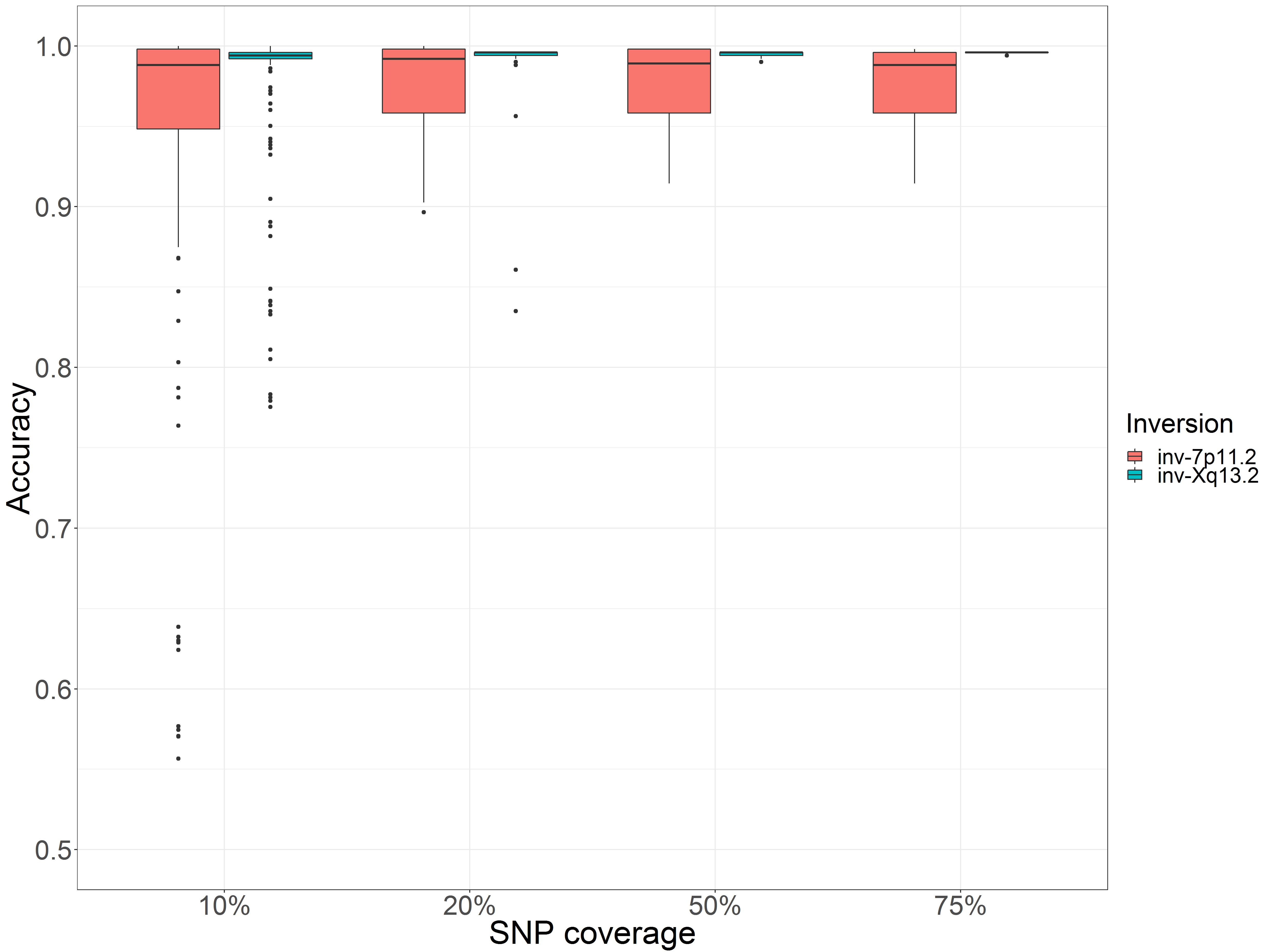

Supplement: S9 Fig — 200 random sets of SNPs were selected at each SNP coverage from the original dataset. (JPG) [file pgen.1008203.s011.jpg]

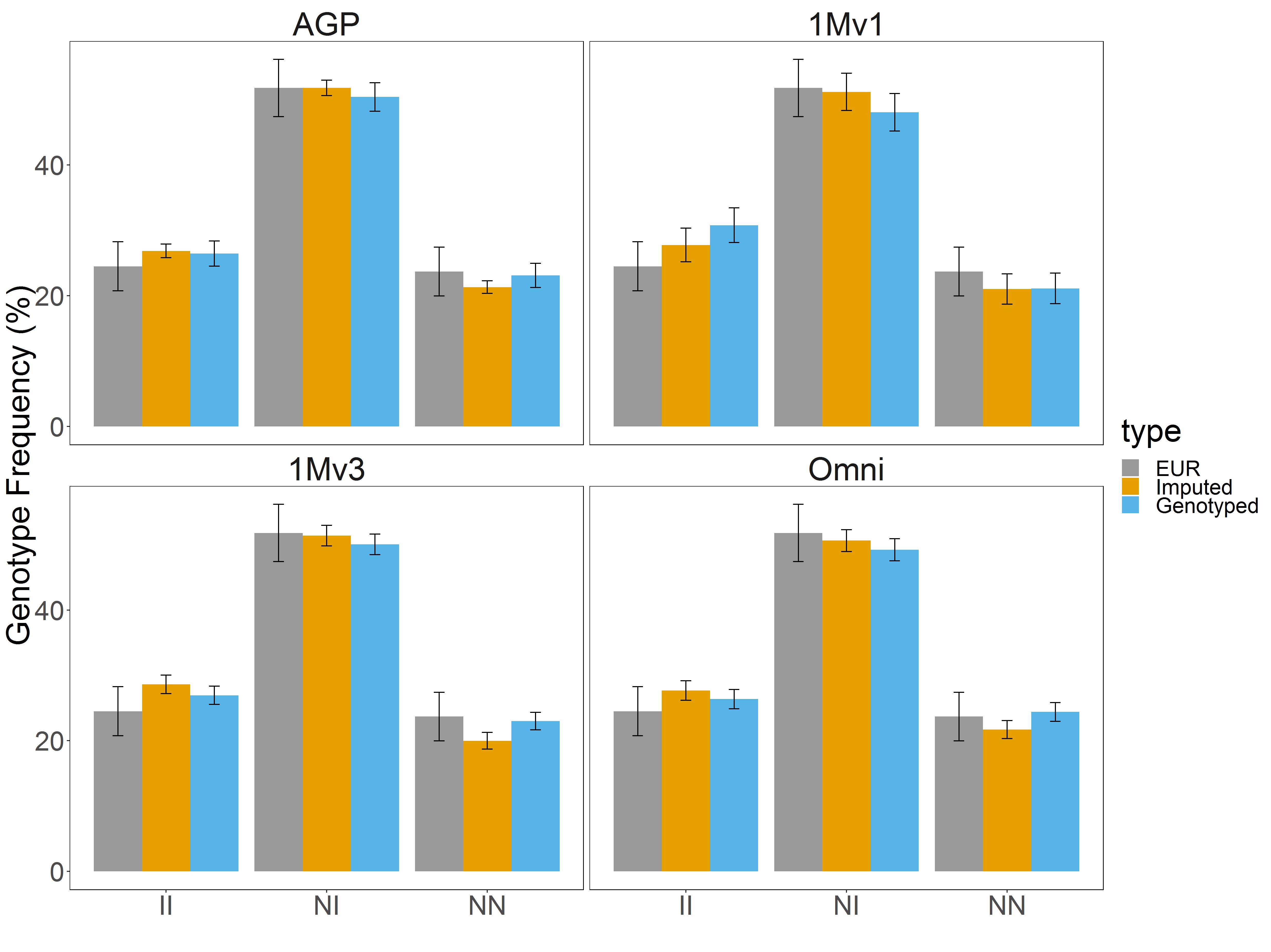

Supplement: S10 Fig — EUR is the frequency in the European individuals of the 1000 Genomes Project. Error bars include the 95% confidence interval of the estimated frequencies. (JPG) [file pgen.1008203.s012.jpg]

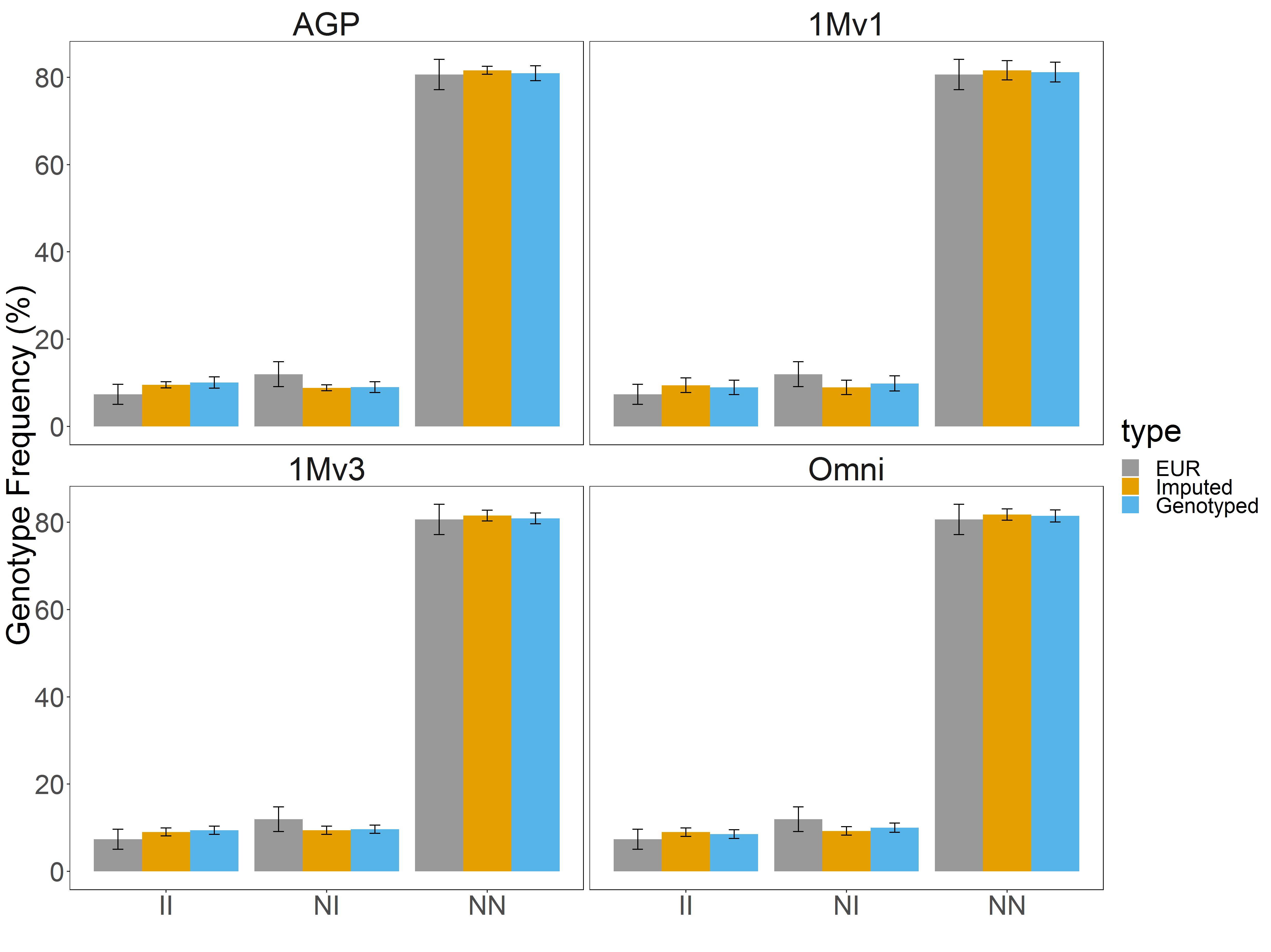

Supplement: S11 Fig — EUR is the frequency in the European individuals of the 1000 Genomes Project. Error bars include the 95% confidence interval of the estimated frequencies. (JPG) [file pgen.1008203.s013.jpg]
