# Supplementary material for: scoreInvHap: Inversion genotyping for genome-wide association studies
Source: PLoS Genet. 2019 Jul 3;15(7):e1008203. doi: 10.1371/journal.pgen.1008203 (PMC6608898; doi:10.1371/journal.pgen.1008203)
Supplement: S1 Table — (DOCX) [file pgen.1008203.s014.docx]

| ***scoreInvHap* Name** | **Locus** | **Original Name** | **Coordinates** | **Num. SNPs** |
| --- | --- | --- | --- | --- |
| inv1_004 | 1p22.1 | INV_delly_INV00005081 | chr1:92,131,841-92,132,615 | 6 |
| inv1_008 | 1q31.3 | INV_delly_INV00008143  HsInv0004 | chr1:197,756,784-197,757,982 | 5 |
| inv2_002 | 2p22.3 | CINV_delly_INV00056184 | chr2:33,764,554-33,765,272 | 6 |
| inv2_013 | 2q22.1 | HsInv0040 | chr2:139,004,949-139,009,203 | 13 |
| inv3_003 | 3q26.1 | HsInv1122 | chr3:162,545,362-162,547,641 | 6 |
| inv6_002 | 6p21.33 | HsInv0058 | chr6:31,009,222-31,010,095 | 5 |
| inv6_006 | 6q23.1 | INV_delly_INV00089348 | chr6:130,848,198-130,852,318 | 12 |
| inv7_003 | 7p14.3 | CINV_delly_INV00092121 | chr7:31,586,765-31,592,019 | 11 |
| inv7_005 | 7p11.2 | HsInv0286 | chr7:54,302,450-54,376,389 | 180 |
| inv7_011 | 7q11.22 | HsInv1053 | chr7:70,426,185-70,438,879 | 10 |
| inv7_014 | 7q36.1 | CINV_delly_INV00099507 | chr7:151,010,030-151,012,107 | 5 |
| inv8_001 | 8p23.1 | HsInv0501 | chr8:8,055,789-11,980,649 | 13,411 |
| inv11_001 | 11p12 | CINV_delly_INV00016131 | chr11:41,162,296-41,167,044 | 7 |
| inv11_004 | 11q13.2 | CINV_delly_INV00018357 | chr11:66,018,563-66,019,946 | 5 |
| inv12_004 | 12q13.11 | INV_delly_INV00022081 | chr12:47,290,470-47,309,756 | 43 |
| inv12_006 | 12q21.2 | CINV_delly_INV00023088 | chr12:71,532,784-71,533,816 | 4 |
| inv14_005 | 14q23.3 | CINV_delly_INV00029966 | chr14:65,842,304-65,843,165 | 4 |
| inv17_007 | 17q21.31 | HsInv0573 | chr17:43,661,775-44,372,665 | 3637 |
| inv21_005 | 21q21.3 | INV_delly_INV00066155  HsInv0045 | chr21:28,020,653-28,021,711 | 11 |
| invX_006 | Xq13.2 | HsInv0396 | chrX:72,215,927-72,306,774 | 135 |

*scoreInvHap* Name: Code names of inversion for *scoreInvHap* call. Original Name: 1000 Genomes coding starts by CINV_delly or INV_delly; InvFEST coding starts by HsInv. Coordinates: inversion coordinates in human assembly hg19. Num. SNP: Number of SNPs with a MAF > 0.05 in the European individuals of the 1000 Genomes Project Phase 3.
