# Supplementary material for: scoreInvHap: Inversion genotyping for genome-wide association studies
Source: PLoS Genet. 2019 Jul 3;15(7):e1008203. doi: 10.1371/journal.pgen.1008203 (PMC6608898; doi:10.1371/journal.pgen.1008203)
Supplement: S2 Table — (DOCX) [file pgen.1008203.s015.docx]

| **Data type** | **Dataset** | **Number of samples** | **Number of SNPs** | | | | |
| --- | --- | --- | --- | --- | --- | --- | --- |
|  |  |  | **Total** | **8p23.1** | **17q21.31** | **7p11.2** | **Xq13.2** |
| **WGS data** | 1000 Genomes European samples | 503 | 25.08m | 170.5k | 21.25k | 2559 | 2263 |
| **SNP data** | AGP | 6951 | 947k | 1833 | 185 | 16 | 12 |
|  | SSC 1Mv1 | 1174 | 1.05m | 1878 | 214 | 17 | 16 |
|  | SSC 1Mv3 | 3836 | 1.20m | 2084 | 235 | 22 | 22 |
|  | SSC Omni | 3437 | 2.44m | 5272 | 416 | 30 | 23 |
|  | 4 Imputed Datasets^1^ | AGP/3-SSC^2^ | 39.64m | 83.2k | 9.24k | 1278 | 831 |
| **Exome data** | Gallagher | 75 | 5.15m | 4449 | 918 | 0 | 7 |
|  | Aberdeen | 387 | 16.38m | 4449 | 918 | 0 | 7 |

1. All 4 datasets were imputed using the same reference panel on the same number of SNPs.

2. Imputed datasets contain the same individuals than original SNP arrays.
