# Supplementary material for: scoreInvHap: Inversion genotyping for genome-wide association studies
Source: PLoS Genet. 2019 Jul 3;15(7):e1008203. doi: 10.1371/journal.pgen.1008203 (PMC6608898; doi:10.1371/journal.pgen.1008203)
Supplement: S3 Table — Table contains the mean and SD runtime in seconds of 10 independent calls. (DOCX) [file pgen.1008203.s016.docx]

| **Method** | **Mean Time (s)** | **SD** |
| --- | --- | --- |
| **invClust** | 134.7 | 0.96 |
| **PFIDO** | 83.7 | 1.46 |
| **scoreInvHap** | 395.7 | 1.03 |
| **scoreInvHap parallel (16 cores)** | 66.2 | 1.67 |
