# Supplementary material for: scoreInvHap: Inversion genotyping for genome-wide association studies
Source: PLoS Genet. 2019 Jul 3;15(7):e1008203. doi: 10.1371/journal.pgen.1008203 (PMC6608898; doi:10.1371/journal.pgen.1008203)
Supplement: S4 Table — (DOCX) [file pgen.1008203.s017.docx]

| **Inversion** | **Datasets** | **Inversion Frequencies** | **HW pval** | **Haplotype Frequencies** | **HW pval** |
| --- | --- | --- | --- | --- | --- |
| **7p11.2** | **AGP** | II: 0.265, NI: 0.504, NN: 0.231 | 0.912 | IaIa: 0.037, IaIb: 0.119, IbIb: 0.108, NaIa: 0.131, NaIb: 0.287, NaNa: 0.155,  NaNb: 0.069, NbIa: 0.053, NbIb: 0.034, NbNb: 0.007 | 1.06e-08 |
|  | **1Mv1** | II: 0.308, NI: 0.481, NN: 0.211 | 0.603 | IaIa: 0.027, IaIb: 0.134, IbIb: 0.146, NaIa: 0.135, NaIb: 0.267, NaNa: 0.141,  NaNb: 0.064, NbIa: 0.079, NbIb: 0, NbNb: 0.006 | 2.7e-30 |
|  | **1Mv3** | II: 0.27, NI: 0.5, NN: 0.23 | 0.99 | IaIa: 0.035, IaIb: 0.123, IbIb: 0.111, NaIa: 0.144, NaIb: 0.283, NaNa: 0.162,  NaNb: 0.062, NbIa: 0.021, NbIb: 0.053, NbNb: 0.006 | 0.619 |
|  | **Omni** | II: 0.264, NI: 0.492, NN: 0.244 | 0.675 | IaIa: 0.04, IaIb: 0.12, IbIb: 0.103, NaIa: 0.132, NaIb: 0.243, NaNa: 0.14,  NaNb: 0.088, NbIa: 0.042, NbIb: 0.075, NbNb: 0.016 | 0.81 |
| **Xq13.2** | **AGP** | II: 0.101, NI: 0.09, NN: 0.809 | 0.794 | II: 0.101, NaI: 0.007, NaNa: 0.041, NaNb: 0.001, NaNc: 0.024, NbI: 0.011,  NbNb: 0.075, NbNc: 0.048, NcI: 0.072, NcNc: 0.62 | 0.722 |
|  | **1Mv1** | II: 0.09, NI: 0.098, NN: 0.812 | 0.683 | II: 0.09, NaI: 0.012, NaNa: 0.043, NaNb: 0, NaNc: 0.048, NbI: 0.012, NbNb: 0.064, NbNc: 0.069, NcI: 0.074, NcNc: 0.588 | 0.158 |
|  | **1Mv3** | II: 0.095, NI: 0.097, NN: 0.809 | 0.597 | II: 0.095, NaI: 0.007, NaNa: 0.04, NaNb: 0.006, NaNc: 0.036, NbI: 0.011,  NbNb: 0.067, NbNc: 0.063, NcI: 0.079, NcNc: 0.597 | 0.998 |
|  | **Omni** | II: 0.085, NI: 0.1, NN: 0.814 | 0.928 | II: 0.085, NaI: 0.004, NaNa: 0.029, NaNb: 0, NaNc: 0.036, NbI: 0.023,  NbNb: 0.074, NbNc: 0.058, NcI: 0.073, NcNc: 0.619 | 1.94e-07 |

HW pal: *P*-value of test of Hardy-Weinberg equilibrium for multiple alleles
