# Supplementary material for: scoreInvHap: Inversion genotyping for genome-wide association studies
Source: PLoS Genet. 2019 Jul 3;15(7):e1008203. doi: 10.1371/journal.pgen.1008203 (PMC6608898; doi:10.1371/journal.pgen.1008203)
Supplement: S5 Table — (DOCX) [file pgen.1008203.s018.docx]

| **Inversion** | **Dataset** | **Genotyped Data** | **Imputed Data** |
| --- | --- | --- | --- |
| **7p11.2** | AGP | 1/513 (0.2%) | 13/2233 (0.6%) |
|  | 1Mv1 | 7/561 (1.2%) | 7/566 (1.2%) |
|  | 1Mv3 | 0/1760 (0%) | 14/1763 (0.8%) |
|  | Omni | 0/1559 (0%) | 10/1599 (0.6%) |
| **Xq13.2** | AGP | 0/489 (0%) | 0/2215 (0%) |
|  | 1Mv1 | 0/505 (0%) | 0/566 (0%) |
|  | 1Mv3 | 0/1680 (0%) | 0/1761 (0%) |
|  | Omni | 4/1080 (0.4%) | 0/1596 (0%) |
