# Supplementary material for: scoreInvHap: Inversion genotyping for genome-wide association studies
Source: PLoS Genet. 2019 Jul 3;15(7):e1008203. doi: 10.1371/journal.pgen.1008203 (PMC6608898; doi:10.1371/journal.pgen.1008203)
Supplement: S6 Table — (DOCX) [file pgen.1008203.s019.docx]

| Autism |  |  |  |
| --- | --- | --- | --- |
| Name | Sample size | Description | EGA identifier |
| UK10K_NEURO_ASD_SKUSE | 361 | This sample set of UK origin consists of clinically identified subjects with Autism Spectrum Disorders, mostly without intellectual disability (*i.e.* Verbal IQ > 70). The subjects represent children and adults with Autism, Asperger syndrome or Atypical Autism, identified according to standardized research criteria (ADI-algorithm, ADOS). A minority has identified comorbid neurodevelopmental disorders (*e.g.* ADHD). Family histories are available, with measures of broader phenotype in first-degree relatives. | EGAS00001000114 |
| UK10K_NEURO_ASD_BIONED | 84 | The BioNED (Biomarkers for Childhood onset neuropsychiatric disorders) study has been carrying out detailed phenotypic assessments evaluating children with an autism spectrum disorder. These assessments included ADI-R, ADOS, neuropsychology, EEG etc. | EGAS00001000111 |
| UK10K_NEURO_ASD_MGAS | 100 | The MGAS (Molecular Genetics of Autism Study) samples are from a clinical sample seen by specialists at the Maudsley hospital and who have had detailed phenotypic assessments with ADI-R and ADOS. | EGAS00001000113 |
| UK10K_NEURO_IMGSAC | 115 | The IMGSAC data set represents an international collection of families containing children ascertained for ASDs (autism spectrum disorders). The affected individuals have been phenotyped using the ADI-R and ADOS instruments. Individuals with a past or current medical disorder of probable etiological significance or TSC have been excluded. Where possible, karyotyping has been performed on one affected individual per family to exclude Fragile X syndrome. Many of the samples have been genotyped, using the Affymetrix 10k and Illumina 1M platforms. All samples to be included in the current study are of UK origin. | EGAS00001000120 |
| UK10K_NEURO_ASD_GALLAGHER | 78 | This is an Irish sample set of individuals with ASD (approximately 50% with comorbid intellectual disability). Individuals have been diagnosed with ADI/ADOS, measures of cognition/adaptive function. They represent a more severe, narrowly defined cohort of ASD subjects. Family histories are available for some with measures of broader phenotype. | EGAS00001000112 |

| Schizophrenia |  |  |  |
| --- | --- | --- | --- |
| Name | Sample size | Description | EGA identifier |
| UK10K_NEURO_IOP_COLLIER | 190 | This data set is comprised of samples from 3 different studies:  The Genetics and Psychosis (GAP) set consists of samples from subjects with schizophrenia, ascertained as a new-onset sample. This set is of UK origin, with data on cognition, brain imaging and other endophenotypes.  The Maudsley twin series consists of probands ascertained from the Maudsley Twin Register, defined as patients of multiple birth who had suffered psychotic symptoms. This set is of UK origin, with data on cognition, brain imaging and other endophenotypes, with DNA available from an MZ or DZ affected or unaffected co-twin.  The Maudsley family study (MFS) consists of over 250 families who have a history of schizophrenia or bipolar disorder. Within the Maudsley Family Study, biological markers of psychosis include neuropsychological tests, Evoked Response Potentials Tests (ERPs), MRI scans, dermatoglyphics and eye tracking. Early risk factors for psychosis and clinical symptoms are also investigated. This set is of UK origin, with DNA available from both affected and unaffected relatives in many of the probands. | EGAS00001000110 |
| UK10K_NEURO_UKSCZ | 631 | These samples have been collected from throughout the UK and Ireland. The samples fall into two main categories. A proportion of these are cases with a positive family history of schizophrenia, either collected as sib-pairs or from multiplex kindred's. The second group consists mainly of samples that have been systematically collected within South Wales and in addition to full diagnostic work up have undergone detailed cognitive testing. All samples have obtained a DSM IV diagnosis of schizophrenia or schizoaffective disorder. | EGAS00001000123 |
| UK10K_NEURO_ABERDEEN | 395 | This sample set comprises cases of schizophrenia with additional cognitive measurements, collected in Aberdeen, Scotland. | EGAS00001000109 |
| UK10K_NEURO_EDINBURGH | 247 | This sample set consists of subjects with schizophrenia recruited from psychiatric in-patient and out-patient facilities in Scotland. All diagnoses are based on standard research procedures and family histories are available. Patients have IQ > 70 and the cohort includes the following groups: 100 cases with detailed clinical, cognitive and structural and functional neuroimaging phenotypes; 138 familial cases who are the probands of families where DNA has been collected from other affected members; 162 unrelated individuals. In most cases patients and their families may be re-contacted to take part in further studies. | EGAS00001000117 |
| UK10K_NEURO_GURLING | 51 | This sample set consists of DNA from multiply affected schizophrenia families. The families have been diagnosed using the SADS-L clinical instrument which gives diagnoses at the probable level of the research diagnostic criteria (RDC). In addition all diagnoses are available using DSMIIIR criteria. These criteria are widely accepted as being valid and reliable for the diagnosis of schizophrenia. All families have been collected to ensure that they are uni-lineal for transmission of schizophrenia, i.e. they have only one affected parent with schizophrenia, or a relative of only one transmitting or obligate carrier parent with schizophrenia. Families with bi-lineal transmission of schizophrenia (i.e. with both parents being affected) were not sampled for this study. All families have multiple cases of schizophrenia and related disorders. The families have been selected to ensure there are no cases of bipolar disorder within them and that they do not contain bipolar disorder in any relatives on either side of the family. | EGAS00001000225 |

| Controls |  |  |  |
| --- | --- | --- | --- |
| Name | Sample size | Description | EGA identifier |
| National Blood Service (NBS) Cohort | 2737 | Healthy blood donors recruited from the United Kingdom Blood Service (UKBS) | EGAS00000000028 |
| 1958 British Birth Cohort | 2930 | 1958 Birth Cohort (1958BC) obtained from EBV-transformed cell lines from individuals born in England, Wales and Scotland during one week in 1958 | EGAS00000000028 |

Descriptions for case studies were obtained from UK10K website (https://www.uk10k.org/studies/neurodevelopment.html[**https://www.uk10k.org/studies/neurodevelopment.html**](https://www.uk10k.org/studies/neurodevelopment.html)). Description for control studies were obtained from (UK IBD Genetics Consortium et al. 2009)
